# Potentially Harmful Elements Associated with Dust of Mosques: Pollution Status, Sources, and Human Health Risks

**DOI:** 10.3390/ijerph20032687

**Published:** 2023-02-02

**Authors:** Bassam Tawabini, Mubarak Al-Enazi, Mansour A. Alghamdi, Ashraf Farahat, Ahsan M. Shemsi, Marwan Y. Al Sharif, Mamdouh I. Khoder

**Affiliations:** 1 Department of Geosciences, College of Petroleum Engineering & Geosciences, King Fahd University of Petroleum & Minerals, Dhahran 31261, Saudi Arabia; 2 Department of Environmental Sciences, Faculty of Meteorology, Environment and Arid Land Agriculture, King Abdulaziz University, P.O. Box 80208, Jeddah 21589, Saudi Arabia; 3 Department of Physics, College of Engineering and Physics, King Fahd University of Petroleum & Minerals, Dhahran 31261, Saudi Arabia; 4 Environmental Chemistry and Analytical Laboratories Section, Center for Environment and Marine Studies, Research Institute, King Fahd University of Petroleum and Minerals, Dhahran 31261, Saudi Arabia; 5 Air Pollution Research Department, Environment and Climate Change Research Institute, National Research Centre, El Behooth Str., Dokki, Giza 12622, Egypt

**Keywords:** trace elements, particulate matter, AC filters, mosques, Saudi Arabia

## Abstract

Potentially harmful elements (PHEs) associated with dust generated from anthropogenic sources can be transported into mosques and deposited on the filters of the air-conditioners (AC); thereby, children and adults are exposed to such PHEs while visiting mosques. Data dealing with the assessment of PHEs pollution and its human health risk in mosques dust in Saudi Arabia are scarce. Therefore, this work aims to examine the levels and pollution status of PHEs in AC filter dust (ACFD) of mosques and their associated human health risk in three Saudi cities: Jubail, Jeddah, and Dammam metropolitan. A similar concentration pattern of PHEs is observed in three cities’ mosques with noticeably higher concentrations than both global crustal and local background values for Zn, Cu, Pb, As, and Cd only. Except for Fe, Al, and Mn, the highest PHEs concentrations were found in Jeddah (1407 mg/kg), followed by Dammam (1239 mg/kg) and Jubail (1103 mg/kg). High PHEs’ concentrations were also recorded in mosques located near workshops and suburban areas compared to urban areas. Based on the spatial pattern, enrichment factor, geo-accumulation index, pollution load index, and ecological risk values, Jubail, Jeddah, and Dammam have shown moderate pollution levels of Cd, As, Pb, and Zn. On the other hand, Cu. Zn, Cu, Cr, Pb, Ni, As, and Cd had degrees of enrichment levels that varied from significantly enriched to extremely highly enriched in the ACFD of the three cities. Heavy pollution is found in Jubail, which posed a higher potential ecological risk than in Jeddah and Dammam. Cd presents the highest ecological risk factors (ER) in the three cities. Carcinogenic and non-carcinogenic risks for children and adults follow the order: Jeddah > Dammam > Jubail, and the ingestion pathway was the main route for exposure. Carcinogenic and con-carcinogenic risks in the mosques of the various studied cities were generally within the acceptable range.

## 1. Introduction

Air quality is one of the major safety and environmental challenges due to its significant contributions to the high global mortality rate [1,2,3]. In particular, indoor air quality is a major concern in urban areas due to the high risks of people’s exposure, proximity to various sources of pollution, and toxic dust [4]. Dust may consist of geologic materials, vehicle emissions, worn car parts, suspended particles from roads, contaminated soil, and airborne particles [5]. Dust may also contain heterogeneous mixtures of harmful chemical-based air pollutants such as polycyclic aromatic hydrocarbons (PAHs), heavy metals, and micro-aerosols that may cause serious environmental and public health problems [6]. Moreover, particulate matters (PMs) are considered serious indoor air-pollutant because of their small size (i.e., PM_10_ and PM_2.5_) and the potential for carrying dangerous heavy metals originating from the exhaust and non-exhaust emissions [7,8]. Heavy metals remain one of the major environmental contaminants largely owing to their toxicity, non-biodegradability, and persistence in the environment [9,10,11]. The extent or level of heavy metal concentration in dust or PM can be assessed by investigating four risk indicators from the sample PM or dust [5]. These include the pollution load index, the geo-accumulation and sensitivity contamination factors, potential ecological risks, and enrichment factors.

The levels, sources, and the index of the ecological risks of heavy metals associated with road dust in India were investigated in the work of Suryawanshi et al. [12]. The results of their study showed that the dust samples were significantly polluted with zinc and lead. In another study, the authors reported high levels of Cd, Cu, Pb, and Zn in equipment dust at seven different playgrounds in Hong Kong due to the proximity to commercial and workshop activities [13]. Additionally, extreme concentrations of Cr, Cu, and Zn were found in ground dust samples in 89 playgrounds [14]. Meanwhile, Fe was found at high levels in the dust of 10 pre-schools in Malaysia [15]. Several approaches have been adopted to identify the sources and types of heavy metal concentration [16].

Several investigations were reported on the safety of air quality in different provinces of Saudi Arabia. These studies focused on the distribution, concentration, and exposure to toxic heavy metals and particulate matter [17]. Moreover, the health and/or ecological risks in different functional areas of Saudi Arabia have been investigated. Some of the functional areas that have been assessed include traffic areas, parking areas, urban areas, industrial, and workshop areas. The level of contamination by heavy metals is largely determined by traffic density, seasonal changes, meteorological conditions, workshop activities, and dust storms [17,18,19,20].

Heavy metal contamination together with its health implications was assessed in the particles in classrooms at the primary schools located in urban, and suburban, areas of Jeddah, Saudi Arabia [21]. For instance, Fe, Mn, and Zn were the dominant heavy metals in the samples studied. Other studies revealed that the traffic density in Riyadh, Saudi Arabia is the major cause of the presence of lead in the atmosphere [22,23]. In another research study, the concentration of heavy metals was assessed at King Faisal University (KFU) campus in the Eastern Province [24]. The study concluded that anthropogenic sources, particularly vehicular movement, largely contributed to the heavy metal contamination and the elimination of leaded gasoline reduced the level of lead in the Eastern Province of Saudi Arabia. It is clear from the above that most of the local studies have mainly focused on heavy metal and PMs’ contamination in ambient air quality associated with traffic, workshops, and urban areas, however, none of these studies have assessed the levels of potentially harmful metals deposited on mosques’ AC filters.

Saudi Arabia is a highly populated Muslim country, where people frequently perform their five daily prayers in mosques. Mosques in Islam are built mainly for the regular five times daily prayers for the congregation. In fact, they are used not only for regular prayers but also for special events such as Friday prayers and night prayers during the month of fasting (Ramadan), when the mosques remain open and people would stay longer. The average temperatures in the Eastern and Western provinces of Saudi Arabia range from 26 to 48 °C during March–October and from 18 to 28 °C from November to February, while the relative humidity could reach up to 65% during the summer [25]. These high temperatures and humidity make worshipers in mosques rely on air conditioning (AC) units to cool down the temperature and reduce humidity in mosques. Maintaining AC units through the periodic cleaning of their filters could play a significant role in improving air quality in mosques. However, many of the mosques were built near anthropogenic activities such as roads, workshops, and small industrial facilities which may lead to the escape of harmful pollutants from AC filters and degrade the indoor air quality of these mosques. Suspended particulate matter that has penetrated from the outdoors to the indoor environment, through doors, ventilation systems, windows, and AC filters for fresh air, in addition to the particulate dust raised from indoor sources and resident activities can be deposited and settled on the AC filter. Consequently, particles that have settled on the AC filter will represent all the exposed particles in the interior environment. Therefore, evaluating the pollution levels and health risks of the different pollutants in the settled dust of AC filters is very significant in enhancing our understanding of these pollutants in the indoor environment. In this study, we investigate the pollution status, sources, and human health risks of potentially harmful elements (PHEs) associated with the AC filter dust (ACFD) of mosques, located in urban, suburban, residential, and workshop areas, within the eastern and western regions of Saudi Arabia.

## 2. Materials and Methods

### 2.1. Study Area

**Figure 1:**
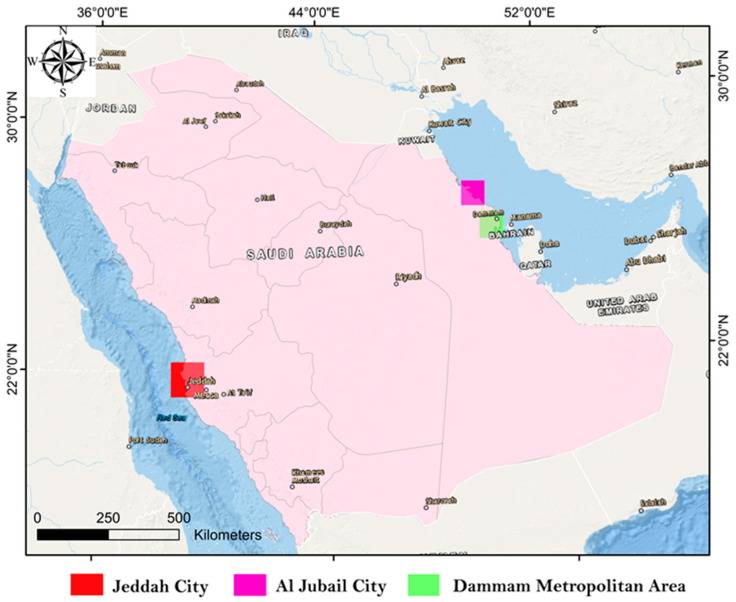
Map of Saudi Arabia illustrating the cities of Jeddah, Jubail, and Dammam.

Saudi Arabia is a relatively large country with an area of about 2.15 M km^2^ and a population of about 36.33 M [26]. In recent years, oil mining activities have increased in various parts of the kingdom thus forcing the government to increase its expenditure in implementing its comprehensive plans. As such, cities across the kingdom have witnessed massive workshop developments, intensive agricultural activities, and an increase in population. In this work, we have focused on three highly populated regions in Saudi Arabia, namely, Jeddah and Jubail cities, and the Dammam Metropolitan area which includes Dammam, Khobar, and Dhahran cities (Figure 1).

Jeddah city (latitude 29.21° N and longitude 39.7° E) is located in the western region of Saudi Arabia (Figure 1), near the eastern coastline of the Red Sea. Jeddah’s current population is about 3.5 million and it accounts for 10% of the entire population of Saudi Arabia. Due to its location along the coastline, Jeddah hosts one of the largest seaports on the Red Sea. The city is also considered the main gateway to the Holy city of Mecca [27], therefore, Jeddah has attracted economic and tourism activities, which have consequently led it to be considered an international economic and trading city. Jeddah’s climate is estimated to be hot desert-like with an average temperature of 28 °C; in summer, the temperature can reach up to 45 °C. The city has rainfall on an annual basis with an average of 61 mm precipitation [28].

Jubail city (latitude 27.39° N and longitude at 49. 70° E) is located in the eastern region of Saudi Arabia (Figure 1), on the Arabian Gulf (AG). Jubail’s current population is about 684,531 and it occupies a landmass of 1016 km^2^ [29]. Jubail hosts some of the largest petrochemical companies in the world and about 172,273 workshops and industrial firms. Apart from the petrochemical plants, the city is also a hub for plastic and fertilizer manufacturers as well as oil- and gas-based ventures. Climatically, Jubail’s summers are arid and long, while winters are dry and cool. Over the course of the year, the temperature varies from 10 to 45 °C. The city has minimal rainfall on an annual basis with an average of 5 mm precipitation [30].

The Dammam metropolitan area (latitude 26.42° N and longitude at 50.08° E) is the largest area in the Eastern Province of Saudi Arabia (Figure 1). The metropolitan area population is about 1,305,000 and it occupies a landmass of 2800 km^2^ [31]. It lies on the west coast of the AG and forms a larger metropolitan and industrial complex with Dammam, Khobar, and Dhahran cities. The summers in Dammam are long, hot, humid, and arid; while the winters are cool, dry, and windy; and it is mostly clear year-round. Over the year, the temperature typically varies from 11 to 43 °C. The area has minimal rainfall on an annual basis with an average of 5 mm precipitation [32].

### 2.2. Sample Collection and Preparation

Air-conditioner filter dust (ACFD) samples, which represent the whole of the exposed particles which have infiltrated from the exterior to interior environment as well as being emitted from indoor activities, were collected from mosques in urban, suburban, residential, and workshop areas, located in Jubail and Jeddah cities and the Dammam metropolitan area, Saudi Arabia (Figure 1). ACFD samples of mosques in the three different cities were mostly collected during 2021. Only 6 samples were collected from Jeddah in 2020. The dust samples were collected from the AC filters of the different mosques after the extensive use of the ACs during the hot weather, from mid-spring to mid-autumn season (7 months). A plastic brush, clean polythene sheets, and airtight polyethylene bags were utilized for the collection of the ACFD samples. The deposited dust on ACF materials was removed in a closed room to ensure proper collection of the deposited dust samples on the filters. In addition, the ACFD particles were trapped slowly due to the small size of the deposited particles on the ACFD that tend to resuspend with ease [33]. The collected ACFD samples were stored in clean, labeled polyethylene bags, transported to the lab, and air-dried at room temperature, and thereafter the coarse impurities were separated using a 1.0 mm mesh nylon sieve. The residue of the dust samples was homogenized, sieved via a 38-μm sieve size, and preserved in small self-sealing plastic bags for PHEs analysis.

### 2.3. Sampling Analysis

The digestion of the sample was carried out following the USEPA 3050 B modified method [34]. About 0.200 mg of the ACFD sample was digested in 5 mL of aqua regia (3:1 ultrapure HCl: HNO_3_) in a 50 mL digestion polypropylene tube at 95 °C on a hot block for two hours. Later, 3 mL, of 30% hydrogen peroxide was added in portions and further heated for half an hour. The resulting digested solution was filtered, and the volume was increased to 50 mL with 1% HNO_3_ solution.

The analysis of PHEs was carried out following USEPA method 6010 [35] on a Perkin Elmer DV 8000 Dual View inductively coupled optical emission spectrometer (ICP-OES). The calibration standards were prepared from CPI mixed standards from 10.0 μg/L to 1000 µg/L concentration. The samples were further diluted appropriately in 1% nitric acid in case the concentration of the analyte exceeded the calibration range. For quality assurance over a batch of 20 samples, 2 samples were duplicated (10%) and 1 sample was spiked, and a spiked duplicate (5%), and analyzed. The relative percent difference was <10% for the duplicate samples and recoveries of the spiked samples were between 92% to 106%. To validate the analysis, a NIST-certified reference material (CRM) 2702 was also digested and analyzed with these samples. The recovery of the NIST CRM was 88% to 104%.

### 2.4. Pollution Characteristics

Various contamination indices including Enrichment factors (EFs), Geo-accumulation index (I_geo_), Contamination factor (C_f_), Contamination degree (C_d_), Pollution load index (PLI), and Potential ecological risk assessment were used to assess the contamination levels of the PHEs associated with the ACFD of the mosques.

#### 2.4.1. Enrichment Factors (EFs)

The enrichment factors (EFs) are a parameter used to examine the level of the anthropogenic effect of PHEs in samples to distinguish between the anthropogenic origins of PHEs and their natural sources [36,37]. Enrichment factors for PHEs in the ACFD of mosques were calculated using Equation (1):

(1) $$\mathrm{EF}=\frac{{\left(C_{n}{/C}_{\mathrm{ref}}\right)}_{\mathrm{sample}}}{{\left(B_{n}{/B}_{\mathrm{ref}}\right)}_{\mathrm{background}}}$$

where C_n_ and B_n_ are the concentration of the target PHEs in the ACFD sample and background (crustal and local soil), respectively. C_ref_ and B_ref_ are the concentration of the reference PHEs in the ACFD sample and background, respectively. Elements such as Al, Fe, Mn, Ti, and Zr, commonly conventional tracers, are usually selected as reference elements in the calculation of EFs for PHEs to detach their anthropogenic sources from their natural origin [38,39]. Although Fe is naturally abundant and has very little effect from anthropogenic sources [40,41], Al is selected as the reference element in EF calculation in the present study. The concentrations of the background PHEs values are taken from the continental crust composition [42,43,44] and the local background soil values [45]. The degree of PHEs enrichment is classified into six pollution classes as shown in Table S1 (Supplementary Materials).

#### 2.4.2. Geo-Accumulation Index (I_geo_)

The Geo-accumulation index (I_geo_) is used to determine the contamination levels by comparing the values of PHEs measured in the ACFD to the background values [46,47]. It evaluates the PHEs pollution in terms of several enrichment classes as listed in Table S1. I_geo_ for mosque dust was calculated using Equation (2):

(2) $$I_{\mathrm{geo}}{=\log}_{2}\;\left(\frac{C_{n}}{1{.5B}_{n}}\;\right)$$

where C_n_ is the measured concentration of the PHEs in the ACFD samples and B_n_ is the PHEs geochemical background value in the earth’s crust and local background soil values. The concentrations of the background PHEs values are taken from the continental crust composition [42,43,44] and local background soil values [45]. The constant 1.5 was used to reduce the impact of probable differences in the background values due to lithological variation in the sediments [48].

#### 2.4.3. Contamination Factor (C_f_) and Contamination Degree (C_d_)

The contamination factor (C_f_) and contamination degree (C_d_) [39] is used to assess the contamination level of PHEs in dust [49]. The C*_f_* index was computed as the ratio of the element value in the ACFD sample to its background concentrations (Equation (3)) [50]:

(3) $$C_{f}=\frac{\left(C_{n\;\mathrm{Sample}}\right)}{\left(C_{n\;\mathrm{Background}}\right)}$$

Meanwhile, the C_d_ was calculated using Equation (4) which considers the sum of C_f_ for PHEs in the ACFD samples:

(4) $$C_{d}=\sum C_{f}$$

The four categories of C_f_ and C_d_ are listed in Table S1 [51,52].

#### 2.4.4. Pollution Load Index (PLI)

The pollution load index (PLI) is used to evaluate the co-contamination of multiple PHEs and can suggest pollution trends over time and make a comparison among different regions [53,54] and can be calculated using Equation (5) [55,56]:

(5) $$\mathrm{PLI}={\left(C_{f1}\times C_{f2}\times C_{f3}\times C_{f4}\times \ldots \times C_{n}\right)}^{\left(\frac{1}{n}\right)}$$

where C_F_ is the contamination factor calculated as described in Equation (3) and n is the number of measured PHEs in the ACFD samples. PLI category standards are listed in Table S1 [53,57,58].

#### 2.4.5. Potential Ecological Risk Assessment

The potential ecological risk index [59] is used to estimate the potential ecological risk for the environment [60]. This index is widely used in contamination evaluations involving a large number of elements [61,62]. The potential ecological risk factor (ER) for the given element was calculated using Equation (6) [63]:

(6) $$\mathrm{ER}=\frac{C_{n}}{C_{b}}\mathrm{TRF}$$

where C_n_ is the concentration of the target elements in the ACFD samples, C_b_ is the background (crustal and local soil) value of the target elements, and TRF is the toxic response factors for the elements which are: As (10), Cd (30), Cr (2), Cu (5), Ni (5), Mn (1), Pb (5), and Zn (1) [59,62,64]. The potential toxicity risk index (ERI) is the sum of the individual ER values (Equation (7)) and is used to evaluate the contamination that is generated by all the measured PHEs simultaneously in the ACFD samples. The values of the ER and ERI grading standard are listed in Table S1.

(7) ERI=∑ER

### 2.5. Human Health Risk Assessment

The health risk assessment models [65] are used to assess the non-carcinogenic and carcinogenic health risks for children and adults exposed to PHEs associated with the ACFD of the mosques. During prayers or gatherings in mosques, people are exposed to PHEs via three base exposure routes including direct hand–mouth ingestion, nasal–oral inhalation, and dermal contact. The sum of the risks estimated from these three exposure routes gives the total non-carcinogenic or carcinogenic health risks for this element.

Based on the models set by [65], the average daily dose (ADD) (mg kg^−1^ day^−1^) for children and adults exposed to PHEs in the ACFD of mosques via the ingestion (ADD_ing_), inhalation (ADD_inh_), and dermal (ADD_dermal_) pathways are calculated in (Equations (8)–(10)):

(8) $${\mathrm{ADD}}_{\mathrm{ing}}=\frac{\left(\;C\times \mathrm{IngR}\times \mathrm{EF}\times \mathrm{ED}\;\right)}{\left(\mathrm{BW}\times \mathrm{AT}\right)}\times 10^{-6}$$

(9) $${\mathrm{ADD}}_{\mathrm{inh}}=\frac{(\;C\times \mathrm{InhR}\times \mathrm{EF}\times \mathrm{ED}\;)}{(\mathrm{PEF}\times \mathrm{BW}\times \mathrm{AT})}$$

(10) $${\mathrm{ADD}}_{\mathrm{dermal}}=\frac{\left(\;C\times \mathrm{SA}\times \mathrm{AF}\times \mathrm{ABF}\times \mathrm{EF}\times \mathrm{ED}\;\right)}{\left(\mathrm{BW}\times \mathrm{AT}\right)}\times 10^{-6}$$

After calculating the ADD, the potentially non-carcinogenic risks of PHEs in the mosques’ ACFD were evaluated by computing the hazard quotient (HQ) (Equations (11)–(13)) and hazard index (HI) (Equation (14)). The incremental lifetime cancer risk (ILCR_)_ and the cancer risks (CR) are calculated using Equations (15)–(18) [66,67]:

(11) $${\mathrm{HQ}}_{\mathrm{ing}}=\frac{{\mathrm{ADD}}_{\mathrm{ing}}}{R_{f}D}$$

(12) $${\mathrm{HQ}}_{\begin{array}{l} \mathrm{inh} \end{array}}=\frac{{\mathrm{ADD}}_{\mathrm{inh}}}{R_{f}D}$$

(13) $${\mathrm{HQ}}_{\mathrm{dermal}}=\frac{{\mathrm{ADD}}_{\mathrm{dermal}}}{R_{f}D}$$

(14) $$\mathrm{Hazard}\;\mathrm{index}\;\left(\mathrm{HI}\right){=\mathrm{HQ}}_{\mathrm{ing}}{+\mathrm{HQ}}_{\mathrm{inh}}{+\mathrm{HQ}}_{\mathrm{dermal}}$$

(15) $${\mathrm{ILCR}}_{\mathrm{ing}}=\frac{C\times \left\{{\mathrm{CSF}}_{\mathrm{ingestion}}\times \sqrt[{3}]{\left(\frac{\mathrm{BW}}{70}\right)}\right\}\times \;\mathrm{IngR}\;\times \;\mathrm{EF}\;\times \;\mathrm{ED}}{\mathrm{BW}\;\times \;\mathrm{AT}\;\times 10^{6}\;}\;$$

(16) $${\mathrm{ILCR}}_{\mathrm{inh}}=\frac{C\;\times \left\{{\mathrm{CSF}}_{\mathrm{inhalation}}\times \sqrt[{3}]{\left(\frac{\mathrm{BW}}{70}\right)}\right\}\times \;\mathrm{InhR}\;\times \;\mathrm{EF}\;\times \mathrm{ED}}{\mathrm{BW}\;\times \;\mathrm{AT}\;\times \;\mathrm{PEF}\;}$$

(17) $${\mathrm{ILCR}}_{\mathrm{dermal}}=\frac{C\;\times \left\{{\mathrm{CSF}}_{\mathrm{dermal}}\times \sqrt[{3}]{\left(\frac{\mathrm{BW}}{70}\right)}\right\}\times \;\mathrm{SA}\;\times \;\mathrm{AF}\;\times \;\mathrm{ABF}\;\times \;\mathrm{EF}\;\times \;\mathrm{ED}}{\mathrm{BW}\;\times \;\mathrm{AT}\;\times 10^{6}\;}\;$$

(18) $$\mathrm{Carcinogenic}\;\mathrm{risk}\;\left(\mathrm{CR}\right)={\mathrm{ILCR}}_{\mathrm{ingestion}}+{\mathrm{ILCR}}_{\mathrm{inhalation}}+{\mathrm{ILCR}}_{\mathrm{dermal}}$$

where R_f_D is the reference dose in mg kg^−1^ day^−1^ [68], an assessment of the highest allowable risks to humans via daily exposure by considering a sensitive group (children) during a lifetime. HQ_i_ is the hazard quotient for a single route. HI is the hazards index, non-carcinogenic risk from the different routes, and equal to the sum of HQ_i_ for different exposure routes. The ILCR_ing_, ILCR_inh_, and ILCR_dermal_ are the incremental lifetime cancer risks by ingestion, inhalation, and dermal contact, respectively. C refers to the concentration of the PHEs (mg kg^−1^) in the ACFD in mosques. CSF is the carcinogenic slope factor (mg/kg/day). BW is the body weight (kg). AT is the average life span (years). EF is the exposure frequency (day/year). ED is the exposure duration (years). InhR is the inhalation rate (m^3^/day). IngR is the ingestion rate (mg/day). SA is the dermal surface exposure (cm^2^). AF is the dermal adherence factor (mg/cm^2^/h). ABF is the dermal absorption factor and PEF is the particle emission factor (m^3^/kg). The complete information regarding the values of exposure factors for children and adults used are listed in Tables S1–S3.

There is an insignificant non-carcinogenic risk when HQ and HI ≤ 1, whereas the probability that non-carcinogenic risk occurs when HQ and HI >1 [65,69]. Likewise, ILCR and CR values from 1 × 10^−6^ to 1 × 10^−4^ can be regarded as an acceptable or tolerable level, values above 1 × 10^−4^ are likely to be unacceptable (potentially high risk), values below 1 × 10^−6^ indicate no significant health hazard [46,55,65,66,70].

## 3. Results and Discussion

### 3.1. Concentrations and Distribution of PHEs in ACFD of Mosques

The summary of descriptive statistics on the PHEs concentrations in the ACFD collected from different mosques located in Jubail and Jeddah cities and the Dammam metropolitan area is showed in Table 1. The variations in the standard deviation (SD) and coefficient of variance (CV) for PHEs in the ACFD of the different mosques were observed across the three studied cities. Based on CV values, the variability was classified into four classes: CV values lower than 20% were considered low, CV values that ranged from 21 to 50% were regarded as moderate, the CV values that ranged from 51 to 100% were considered high, and CV values above 100% were deemed extremely high [71]. In the present study, the low CV values for the PHEs in the ACFD suggest a homogenous distribution and dominance of definite types of pollution sources, whereas the moderate to high CV values indicate a heterogeneous PHEs distribution and a notable diversity in the pollutant sources [72,73]. Generally, Cr has extremely high variability in the ACFD of Jeddah mosques (JEMs), Pb is highly variable in the ACFD of JEMs and Jubail mosques (JUMs) as well as the Dammam metropolitan area mosques (DMMs), and Mn and Cr in JUMs and Ni in JEMs are low in variability. Moderate variability is typical for the remaining PHEs in the ACFD of the studied cities’ mosques (Table 1).

Overall, the sequences of the mean concentrations of the PHEs in the ACFD of mosques in each city are as follows: Fe > Al >Zn > Mn > Cu > Pb > Cr > Ni > V > Co > As > Cd in both JUMs and DMMs and Fe > Al > Zn > Mn > Cu > Pb > Cr > V > Ni > Co > As > Cd in JEMs. According to the results, Fe and Al concentrations are the highest in the studied cities’ mosques, whereas As and Cd are the lowest levels. Both Fe and Al are abundant elements in the earth’s crust and are found in the soil [74,75], therefore the highest concentration of these two elements in the ACFD of mosques of the studied cites might suggest that their occurrence could be from natural sources (local soil). Except for Fe, Al, and Mn which are considered crustal elements, the highest total concentrations of the other nine elements (∑Zn, Cu, Cr, Pb, Ni, V, Co, As, and Cd) were found in the ACFD of JEMs followed by DMMs and JUMs. Moreover, the mean values for the total nine elements concentrations were in the following order: 1407 mg/kg (JEMs) > 1239 mg/kg (DMMs) > 1103 mg/kg (JUMs), indicating that these PHEs that accumulated in the ACFD of mosques probably derived from anthropogenic outdoor sources near the mosques, such as vehicular traffic, construction and, destruction activity, waste disposal [76], and the variations in PHEs concentrations of mosques’ dust depends on the intensity of their sources emission in the outdoor surrounding environment, since there are no direct emission sources for these PHEs in the indoor environment. Dust in the indoor environment is a mixture of indoor aerosols, and soil/dust tracked in by foot traffic and ventilation [77]. Outdoor dust/soil contributes up to 45% of the elemental load in indoor dust [78]. Outdoor dust is affected by vehicular traffic emissions such as the wear and tear of tires, diesel fuel and gasoline, lubricating oil grease, bearings’ wear, engine parts, and brake emissions, particularly from high-density traffic areas, and is the main source of elements such as Cd, Cu, Zn, and Ni in indoor environments in that area [79]. It is well known that PHEs’ sources in the indoor environment are natural and anthropogenic, therefore the relatively higher levels of anthropogenic PHEs in the ACFD of JEMs and DMMs than those found in JUMs may likely be attributed to the high population density of these cities with high traffic density and indicates that traffic emissions have made large contributions to PHEs’ pollution [80,81].

The PHEs’ mean concentrations in the ACFD of mosques in the different cities exceeded the global crustal background values [42,43,44] only for Zn, Cu, Pb, As, and Cd. Their mean concentration levels were 10.9, 2.9, 4.1, 2.4, and 5.5 times (JUMs), 11.9, 3.8, 4.3, 2.5, and 6.1 times (DMMs), and 11.9, 3.9, 8.8, 2.1, and 7.2 times (JEMs) higher than those in the global crustal background, respectively (Table 1). Compared with local background soil values in Saudi Arabia, the mean concentration levels of Zn, Cu, Cr, Pb, Ni, As, and Cd in JUMs, DMMs, and JEMs, and both V and Co in DMMs and JEMs only exceeded the local background soil values which were reported in a rural area in Saudi Arabia [45]. The average concentrations of Zn, Cu, Cr, Pb, Ni, As, and Cd were approximately 9.4, 7.9, 1.8, 3.4, 2.0, 1.9, and 2.7 times (JUMs), 10.3, 10.3, 1.2, 3.6, 2.2, 2.0, and 3.0 times (DMMs), and 10.3, 10.6, 2.1, 7.3, 2.3, 1.7, and 3.6 times (JEMs) higher than their concentrations in the local background soil values, respectively. These results indicate that these PHEs in the ACFD of these mosques were strongly affected by anthropogenic sources [62,82,83] and accumulated in varied degrees.

From the comparison of selected measured PHEs, such as Cd, Co, Cu, Pb, and Zn concentrations in the ACFD of mosques in the studied cities with their maximum permissible concentrations (MPC): 3 (Cd), 30 (Co), 100 (Cu), 1500 (Mn), 100 (Pb), and 300 (Zn) mg/kg in soil [84], only Pb in JEMs’ indoor dust and both Cu and Zn in JEMs, DMMs, and JUMs were higher than the MPC and thus might pose significant human health threats [85]. So far, data concerning PHEs associated with mosques’ dust are scarce not only in Saudi Arabia but also around the world. Accordingly, the PHEs’ patterns and levels in the ACFD of JEMs, DMMs, and JUMs were compared with those found in indoor dust from different cities around the world (Table 2). There is a unanimous agreement that As, Co, and Cd are the lowest concentrations PHEs, whereas Al, Fe, Mn, and Zn are the maximum concentrations in indoor dust. The PHEs concentrations in the ACFD of JEMs, DMMs, and JUMs were lower/higher or similar to those found in indoor dust of other cities in the world (Table 2). The observed variations in the PHEs’ concentrations in indoor dust among these cities around the world augment the concept that these PHEs contamination in the indoor environment can be associated with exterior anthropogenic sources diversities such as urbanization activities, traffic density, economic growth, the types of fuels used in different cities, beside the indoor sources such as fuel combustion and smoking, the proximity to the main streets, and the rate of dust infiltration from the outdoor to the indoor environment.

**Figure 2:**
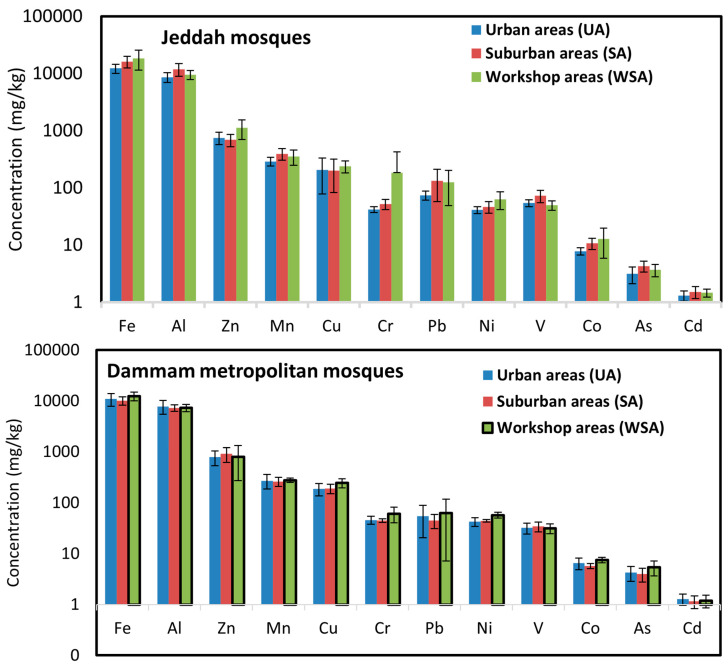

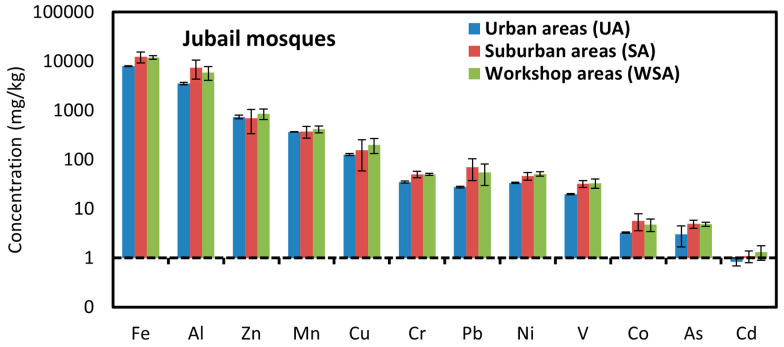
The concentrations of PHEs in ACFD of mosques located in different cities are based on function areas (urban areas-UA; suburban areas-SA; workshop areas-WSA).

The spatial variations of PHEs concentrations in the ACFD collected from mosques in the different functional areas in Jubail and Jeddah cities and the Dammam metropolitan area is shown in Figure 2. The concentrations of all PHEs (except Al, Fe, and Mn) in the ACFD of mosques located in workshop areas (WSA) and suburban areas (SA) were relatively higher than those found in urban areas (UA). Moreover, the mean concentrations of the total nine anthropogenic elements (∑As, Cd, Co, Cr, Cu, Ni, Pb, V, and Zn) in WSA, SA, and UA mosques, respectively, were 1257, 1060, and 994 mg/kg in Jubail, 1268, 1283, and 1165 mg/kg in Dammam metropolitan area, and 1812, 1221, and 1188 mg/kg in Jeddah.

These results indicate that the pattern of the spatial distribution of PHEs concentrations in the ACFD was possibly related to exterior local anthropogenic sources that were found along/near or in the area surrounding the mosques. Moreover, the measured concentration gradient of indoor PHEs between UA, SA, and WSA mosques is not consistent with a single point source in each city but is more likely to be interpreted by local vehicular density in the streets around the sampling sites.

With regard to mosques, the origin of indoor PHEs emissions was similar, as there are no activities in the investigated mosques in the three studied cities. Therefore, the differences in PHEs levels associated with the ACFD in the various mosques are largely related to outdoor sources of PHEs. Infiltration of exterior particulate-bound PHEs emitted from nearby vehicular traffic into mosques may be the principal origin of PHEs in the mosques’ indoor dust. The high emissions from the vehicular density and the maintenance car activities in WSA, beside the heavy traffic along highways roads that run neighboring SA mosques beside building construction and demolition activities as well as waste disposal [33] lead to an increase in the levels of PHEs in the outdoor dust, and consequently accumulation in mosques indoor dust of WSA and SA. Auto repair activities and mechanic workshops are one of the main sources of the increase in the PHEs levels in dust [67,100,101]. Soils quality of automobile workshops was rated as seriously contaminated with PHEs, since several activities such as the servicing of vehicle engines, panel beating of vehicle bodies and scraping of old vehicle bodies, spraying of vehicles, and greasing and oiling of car parts lead to an increase in the contamination by Cd, Cr, Pb, and Zn [68]. Activities such as the repair and/or charging of batteries, in an automobile mechanic village in Ibadan, Nigeria, are known to be sources of Zn and Cd [102].

**Figure 3:**
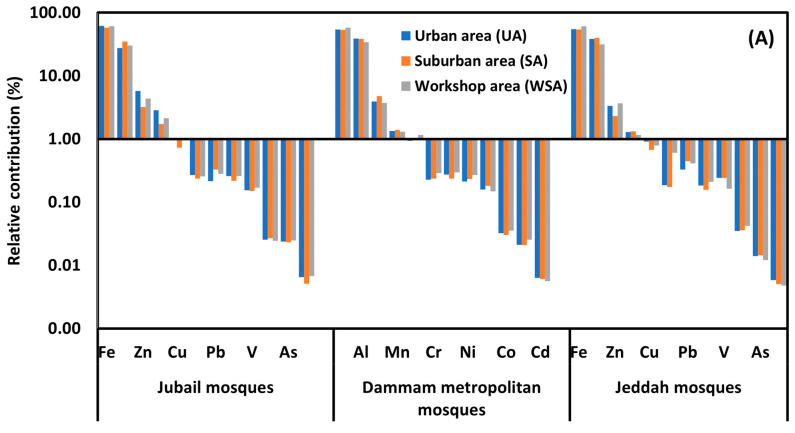

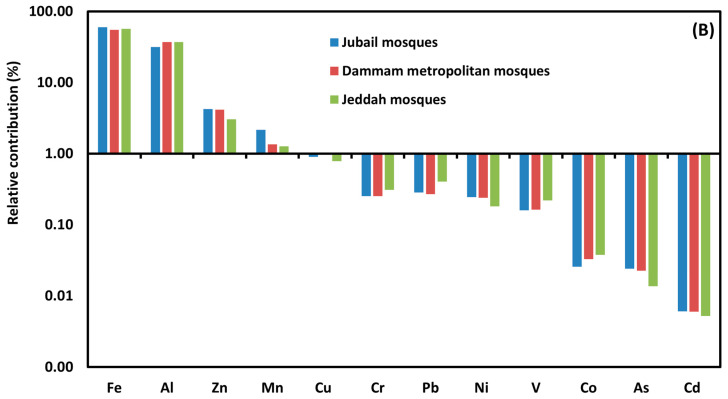
Relative contribution (%) of each PHE to ∑PHEs concentrations in ACFD of mosques located in different functional areas of Jubail and Jeddah cities and Dammam metropolitan area (**A**), and in ACFD of Jubail, Jeddah and Dammam metropolitan area mosques (**B**).

The relative contribution of the individual PHE to the ∑PHEs concentrations in the ACFD of JUMs, DMMs, and JEMs is shown in Figure 3. The distributions of the PHEs in the ACFD of UA, SA, and WSA mosques of Jubail and Jeddah cities and Dammam metropolitan area were similar, with the highest contribution for Al, Fe, Mn, and Zn and the lowest for As, Cd, and Co (Figure 3a). Generally, the spatial variations in the individual PHE distributions concentration in indoor dust, of JUMs, DMMs, and JEMs were quite similar. They follow the decreasing pattern: Fe > Al > Zn > Mn > Cu > Pb > Cr > Ni > V > Co > As and Cd (Figure 3b). This similarity in the PHEs distribution highlights that PHEs in the ACFD of the different mosques do share a common source, and maybe traffic emissions around the mosques are the predominant sources of PHEs. The traffic exhausts, lubricating oil residues, tire wear, asphalt, and brake lining wear are well-known anthropogenic sources of PHEs [37,70,103,104].

### 3.2. PHEs Pollution Characteristics in ACFD of Mosques

#### 3.2.1. Contamination Factors (C_f_) and Degree of Contamination (C_d_)

**Figure 4:**
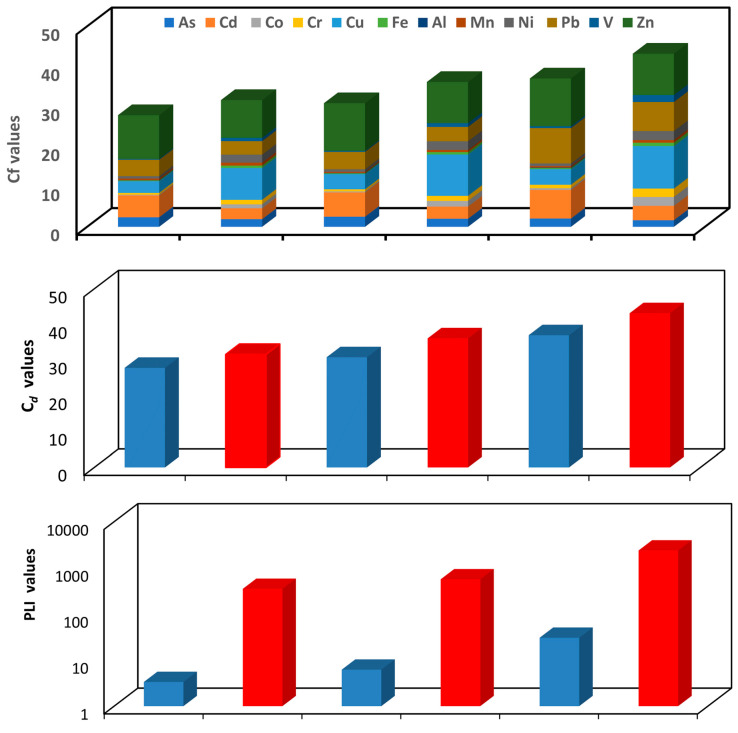

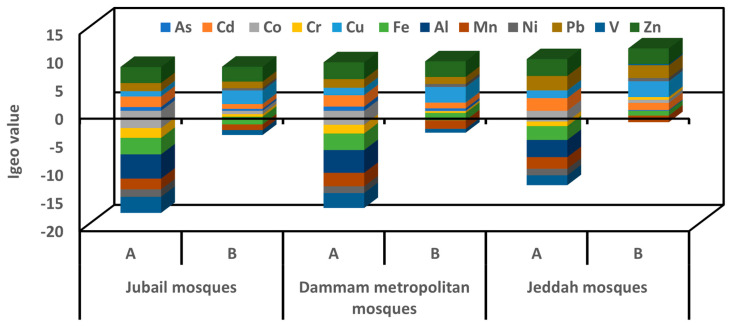
Contamination factor (C_f_), degree of contamination (C_d_), pollution load index (*PLI*), and geo-accumulation index (I_geo_) of PHEs in ACFD of Jubail, Dammam metropolitan, and Jeddah mosques: Based on the global crustal data (A) and local background soil data (B).

Based on the global crustal and the local background soil data, the calculated contamination factors (C_f_) for the PHEs in the ACFD of JUMs, DMMs, and JEMs are shown in Figure 4 and Table S4. They varied from low to very high contamination depending on the classification criteria of C_f_ (Table S5). The calculated C_F_ values for Fe and Mn in the JUMs, DMMs, and JEMs (based on the global crustal and local background soil data), V, Co, Ni, and Cr in both JUMs and DMMs (based on the global crustal date), and V in both JUMs and DMMs (based on the local background soil data) were lower than one, implying low contamination and their origin from soil and resuspended dust. The C_f_ values of As and Cu in the JUMs and As in the DMMs and JEMs (based on the global crustal data) and As, Cd, Co, Cr, and Ni in the JUMs, As, Cd, Co, Cr, and Ni in the DMMs and As, Co, Cr, Ni, and V in the JEMs (based on the local background data) were between one and three, indicating moderate contamination. The C_f_ values of Cd and Pb in the JUMs, Cu, and Pb in the DMMs and Cu in the JEMs (based on the global crustal data) and Pb in both JUMs and DMMs and Cd in the JEMs (based on the local background soil data) were between three and six, suggesting considerable contamination. The C_f_ values of Zn in the JUMs, DMMs, and JEMs and Pb in JEMs (based on the global crustal and the local background soil data), Cu in the JUMs, DMMs, and JEMs (based on the local background soil data) and Cd in both DMMs and JEMs (based on the global crustal data) were higher than six, implying very high contamination. In general, the results of C_f_ evaluation indicated that the Cf values for PHEs in the ACFD of the different JUMs, DMMs, and JEMs had a large variation, with the highest Zn, Pb, Cd, and Cu values. The average concentrations of Zn, Cu, Pb, and Cd, respectively, were 9.4, 7.9, 3.4, and 2.7 times (JUMs), 10.3, 10.3, 3.6, and 3.0 times (DMMs), and 10.3, 10.6, 7.3, and 3.6 times (JEMs) higher than their concentrations in the local background data. Moreover, they were 10.9, 2.9, 4.1, and 5.5 times (JUMs), 11.9, 3.8, 4.3, and 6.1 times (DMMS) and 11.9, 3.9, 8.8, and 7.2 times (JEMs) higher than those in the crustal background data, respectively. Hence, it could be confirmed that the pollution of Cd, Cu, Pb, and Zn was notably influenced by anthropogenic activities.

The degree of contamination (C_d_) analysis of PHEs in the ACFD of the JUMs, DMMs, and JEMs is shown in Figure 4 and Table S4. They varied from a moderate to a strong degree of contamination to a very strong degree of contamination depending on the classification criteria of C_d_ (Table S5). Very high degrees of pollution were noticed in JEMs (based on the global crustal and the local background soil data) and in DMMs (based on the local background soil data), whilst a moderate to a strong degree of contamination was found in the JUMs (based on the global crustal and the local background soil data) and in DMMs (based on the global crustal data). The C_d_ values were 27.9 and 31.6 (JUMs), 30.9 and 36.1 (DMMs), and 37.0 and 43.2 (JEMs) based on the global crustal and local background soil data, respectively. The highest degree of contamination of indoor dust of JEMs than those in both DMMs and JUMs by PHEs may be due to the higher traffic density, which is the main source of these metals in the study areas, in the area around the JEMs than the areas around the DMMs and JUMs.

#### 3.2.2. Pollution Load Index (PLI)

To assess the cumulative contamination load impact by ∑PHEs, the pollution load index (PLI) was calculated. The calculated PLI for the total PHEs in the ACFD of JUMs, DMMs, and JEMs are represented graphically in Figure 4 and listed in Table S4. Depending on the classification criteria of PLI (Table S5), the PLI values in the ACFD collected from all mosques indicated the presence of a cumulative pollution load of PHEs. The highest PLI values were found in JEMs followed by DMMs, and JUMs. The PLI values were 3.35 and 349.9 (JUMs), 6.2 and 563.5 (DMMs), and 30.5 and 2378.5 (JEMs) based on the global crustal and the local background soil data, respectively, suggesting a high pollution load (heavy contamination) of PHEs in all studied mosques. This variety of PHEs pollution levels between the mosques located in the three studied cities almost reflected the effect of anthropogenic activities. Therefore, the highest pollution load of all PHEs in the Jeddah mosques could be due to the impact of high traffic emissions around them. Previous studies have concluded that car emissions are the major source of heavy metals in interior dust [21,105,106,107].

#### 3.2.3. Geo-Accumulation Index (I_geo_)

The I_geo_ values for the PHEs in the ACFD of the JUMs, DMMs, and JEMs are calculated and interpreted based on the global crustal and the local background soil data and are shown in Figure 4 and Table S4. The highest I_geo_ values were found for Cd, Cu, Pb, and Zn whereas the lowest values were found for Co, Cr, Fe, Mn, Ni, and V. According to the criteria of pollution based on I_geo_ value (Table S5), the ACFD was unpolluted (based on the global crustal and the local background soil data) by Fe and Mn in the JUMs, DMMs, and JEMs, by Co, Cr, and V in both JUMs and DMMs (based on the global crustal and the local background soil data), Co, Cr, Ni and V in the JEMs (based on the global crustal data), Ni in both JUMs and DMMs (based on the global crustal data). The ACFD was uncontaminated to moderately contaminated by As (based on the global crustal and the local background soil data) in the JUMs, DMMs, and JEMs, Cu in the JUMs (based on the global crustal data), Cd and Ni (based on the local background soil data) in both JUMs and DMMs, and Co, Cr, Ni, and V (based on the local background soil data) in the JEMs; moderately contaminated by Cd and Pb in JUMs, Cd, Cu, and Pb in the DMMs, and Cu in the JEMs (based on the global crustal data) and Pb in both DMMs and JUMs and Cd in the JEMs (based on the local background soil data); and moderately polluted by Zn (based on the global crustal and the local background soil data) in the JUMs, DMMs, and JEMs, Cu (based on the local background soil data) in the JUMs, DMMs, and JEMs and Cd and Pb (based on the global crustal data) in the JEMs. High I_geo_ values for Zn, Cd, Cu, and Pb in the ACFD of the different studied mosques can be attributed to the increase in the outdoor emissions of these elements that are released from exterior anthropogenic sources, consequently, the increase in their content in indoor dust of mosques. High levels of metal pollution arise not only from growing socio-economic activities that include traffic density but also from the insufficient adequate disposal protocols for many spent products such as paint, oil, grease, fuel, and tires [108,109].

#### 3.2.4. Enrichment Factor (EF)

**Figure 5:**
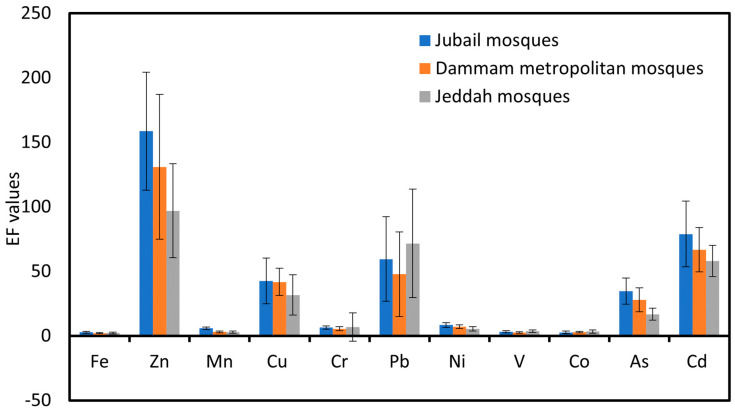
Enrichment factors analysis (EFs) of PHEs in ACFD of Jubail, Dammam metropolitan, and Jeddah mosques.

The calculated enrichment factors (EFs) for measured PHEs in the ACFD of the JUMs, DMMs, and JEMs are represented graphically in Figure 5. The degree of enrichment varied from moderate to extreme enrichment according to the classification criteria of the EF values (Table S5). The mean EF values of PHEs in the ACFD displayed the following decreasing trend: Zn > Cd > Pb > Cu > As > Ni > Cr > Mn > V > Fe > Co in the JUMs, Zn > Cd > Pb > Cu > As > Ni > Cr > Mn > V > Co > Fe in the DMMs, and Zn > Pb > Cd > Cu > As > Cr > Ni > V > Co > Mn > Fe in the JEMs (Figure 5). Based on the calculated EF values for the PHEs in the ACFD of the JUMs, DMMs, and JEMs and their classification criteria, the EF values ranging from two to five (moderate enrichment) were found for Co, Fe, and V in JUMs, DMMs, and JEMs, and Mn in both DMMs and JEMs. EF values from 5 to 20 were detected for Mn in the JUMs, As in the JEMs, and both Cr and Ni in the JUMs, DMMs, and JEMs, suggesting that they were significantly enriched, whilst values from 20 to 40 were found for Cu in the JEMs and As in both JUMs and DMMs, indicating they were strongly enriched. Moreover, EF values higher than 40 were recorded for Cu in both JUMs and DMMs and Zn, Pb, and Cd in the JUMs, DMMs, and JEMs, suggesting that they were extremely enriched. These results indicated that the seven PHEs (As, Cd, Cr, Cu, Pb, Ni, and Zn) in the ACFD of the JUMs, DMMs, and JEMs had degrees of enrichment levels that varied from significantly to extremely highly enriched, indicating that the origin and the accumulation of these PHEs in the ACFD of these mosques was almost completely influenced by the exterior anthropogenic sources. Anthropogenic PHEs such as As, Cd, Cr, Cu, Ni, Pb, and Zn are mainly released from traffic emissions, fossil fuel combustion [21,83,97,110,111,112,113,114,115], greasing oil, and tire wear and tear [75]. Moreover, the corrosive process of the wearing down of the wares, walls, batteries, street safety barriers, lamps, and railings under the effect of weather conditions may lead to an increase in the release of PHEs such as Cd, Cr, Cu, and Zn in urban dust [89,116,117,118,119]. Although Pb was phased out in Saudi Arabia in 2011 [120], it was discovered as extremely enriched in the ACFD-collected samples from all mosques of the three studied cities. This indicates that the historical contamination of Pb can persist in the local environment as a fraction of traffic-produced street dust with its long half-life [21,45,121,122], and can also be resuspended [123,124].

#### 3.2.5. Ecological Risk Assessment

**Figure 6:**
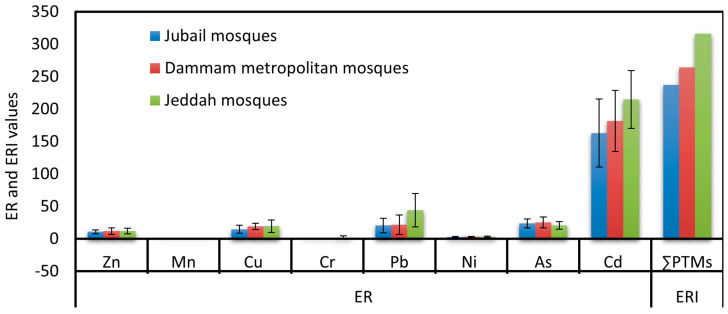
Variation of potential ecological risks for PHEs in ACFD of Jubail, Dammam metropolitan, and Jeddah mosques.

The potential ecological risk factors (ER) for As, Cd, Cr, Cu, Mn, Ni, Pb, and Zn in the ACFD of the JUMs, DMMs, and JEMs were calculated and shown in Figure 6. The pattern of ER values in descending order was Cd > As > Pb > Cu > Zn > Ni > Cr > and Mn in both JUMs and DMMs and Cd > Pb > As > Cu > Zn > Ni > Cr > Mn in JEMs. The highest ER values were 163.1, 181.7, and 214.7 for Cd, 23.9, 25.3, and 20.7 for As, and 20.5, 21.7, and 44.1 for Pb in the JUMs, DMMs, and JEMs, respectively. Depending on the classification criteria of ER (Table S5), As, Cr, Cu, Mn, Ni, and Zn in the JUMs, DMMs, and JEMs and Pb in both JUMs and DMMs showed lower ecological risk (ER < 40). ER values of 40 ≤ ER < 80 were found for Pb in the JEMs, indicating a moderate risk to the ecological environment. On the other hand, Cd showed a higher risk to the ecological environment.

### 3.3. Human Health Risk Evaluation

To assess the health risks for children and adults from exposure to PHEs associated with the ACFD of the JUMs, DMMs, and JEMs mosques, the carcinogenic and non-carcinogenic health risks for both children and adults were calculated via the direct hand–mouth ingestion, nasal–oral inhalation, and dermal contact. The results were evaluated based on the computed values of the hazard quotients (HQs: HQ_ing_, HQ_inh._, HQ_dermal_), hazard index (HI, (∑exposure routes)), incremental lifetime cancer risk (ILCR), and cancer risk (CR, (∑exposure routes)).

#### 3.3.1. Non-Carcinogenic Risk

Based on the concentrations of PHEs associated with the ACFD of the various JUMs, DMMs, and JEMs in Table 1 and the values of exposure factors for children and adults (Tables S1–S3), the HQ_ing_, HQ_inh_, HQ_dermal_, and HI for both children and adults exposed to PHEs were computed and listed in Table 3, Table 4 and Table 5. The values of HQ suggest that HQ_ing_ was most likely to pose a non-cancer risk of more than both HQ_derma_l and HQ_inh_. The values of HQ_ing_, HQ_inh_, and HI for children were, respectively, 9.1, 1.48, and 5.2 (JUMs), and 9.2, 2.8, and 5.5 (DMMs) and 9.4, 2.8, and 5.0 (JEMs) times more than that of adult in the JUMs, DMMs, and JEMs, whereas HQ_dermal_ for adults were 2.1 (JUMs), 2.0 (DMMs), and 2.0 (JEMs) times more than that of children. The increased tendency of children to ingest dust-associated PHEs possibly due to their crawling and hand-to-mouth movements [125,126] led to an increase in the HQ_ing_ and HI values for children more than those for adults; this is consistent with previous research findings [21,67].

The percentage risk contributions of HQ_ing_, HQ_inh_, and HQ_dermal_ to the HI for the total elements (∑PHEs) were 93.2, 2.8, and 4.0% (JUMs), 93.6, 2.4, and 4.0% (DMMs), and 93.2, 2.3%, and 4.5% (JEMs) for the children, respectively. Similarly, the risk contributions of HQ_ing_, HQ_inh_, and HQ_dermal_ to the HI for adults were 52.3, 5.3, and 42.5 in JUMs, 52.7, 4.6, and 42.7% in DMMs, 50.0, 4.2, and 45.9% in JEMs, respectively. Accordingly, ingestion for both adults and children was the main exposure pathway of PHEs associated with the ACFD of the JUMs, DMMs, and JEMs, which is similar to the findings of numerous previous studies [21,127,128].

**Figure 7:**
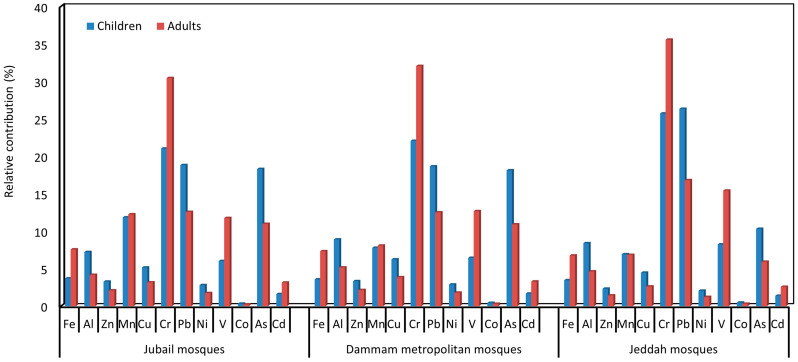
Relative contribution of the individual PHE hazard index (HI) to ∑HI for total PHEs in Jubail, Dammam metropolitan, and Jeddah mosques for children and adults.

According to USEPA (2011), it is well known that the HQs ≥ 1 indicate an unsafe risk (harmful health effects), whereas HQs < 1 suggest an acceptable hazard level. Based on the exposure to PTEs concentrations for children and adults in the JUMs, DMMs, and JEMs, HQ_ing_, HQ_inh_, HQ_dermal_, and HI values for the individual PTE and ∑PTEs were within the safe level (<1) and indicate that there was no non-carcinogenic risk via the various exposure routes (Table 3, Table 4 and Table 5). With regard to the contribution of HI for the individual PHE to the ∑HI for the total measured PHEs (Figure 7), Cr, As, and Pb represent the most predominant elements for children, accounting for 21.0%, 18.3%, and 18.6% (JUMs), 22.4%, 18.1%, and 18.6% (DMMs) and 25.7%, 10.3%, and 26.3% (JEMs) of the values of ∑HI, respectively. Meanwhile, for adults, Cr, Mn, and Pb in JUMs, V, Cr, and Pb in both DMMs and JEMs were the dominant elements, accounting for 30.4%, 12.2, and 12.6% (JUMs), 12.7%, 32.0%, and 12.5% (DMMs) and 15.4%, 35.6%, and 16.8% (JEMs) of the values of ƩHI, respectively. As a result, the probable non-carcinogenic risk from exposure to V, Cr, Mn, As, and Pb, in the indoor dust of JUMs, DMMs, and JEMs cannot be neglected, since any further increase in the anthropogenic activities may raise their contamination levels and hence the health risk. Consequently, PHEs require more continuous and periodic studies in the indoor environment of mosques.

#### 3.3.2. Carcinogenic Risk

Among the measured twelve PHEs, ILCRs for six PHEs (As, Cd, Co, Cr, Ni, and Pb) in the ACFD of JUMs, DMMs, and JEMs were selected and considered in order to assess carcinogenic risk [129] for children and adults. The calculated ILCRs (ILCing, ILCRinh, ILCRdermal) and total cancer risk (CR = ∑ILCRing + ILCRinh + ILCRdermal) are listed in Table 3, Table 4 and Table 5. ILCRing was higher for children than for adults and vice versa for ILCRinh, ILCRdermal, and CR in JUMs, DMMs, and JEMs. The ILCRing for children was 1.4 times more than that for adults, whereas ILCRinh and ILCRdermal for adults were 2.4 and 13.7 times more than that of children in the various mosques. Moreover, the CR for adults was 1.3 times more than that for children, confirming that the exposure to indoor mosques dust can cause more potential health risks for adults than for children. The sequence of ILCRs and CR for children and adults from the exposure to ∑ individual and ∑PHEs in the ACFD of the different mosques was JEMs > DMMs > JUMs. This difference in cancer risks among the mosques of the three different cities suggested the considerable effects of source emissions of PHEs on the health risk. Based on the exposure pathways for children and adults, ILCRs values decreased in the following order: ingestion > dermal contact > inhalation in JUMs, DMMs, and JEMs. The risk contributions of ILCRing, ILCRinh, and ILCRdermal to the CR from the exposure of total measured elements (ƩPHEs) in mosques, respectively, were 95.8, 0.1, and 4.1% (JUMs), 95.8, 0.1, and 4.1% (DMMs), and 95.5, 0.1, and 4.4% (JEMs) for the children and 55.0, 0.1, and 44.9% (JUMs), 54.9, 0.1, and 45.0% (DMMs), and 53.3, 0.1, and 46.6% (JEMs) for adults. Accordingly, ingestion for both adults and children was the main exposure pathway of PHEs associated with the ACFD of the JUMs, DMMs, and JEMs, followed by the dermal and inhalation pathways. This is in agreement with previous studies which reported that the non-dietary ingestion and dermal pathways were responsible for most of the total CR [21,67,75,89,93,127].

**Figure 8:**
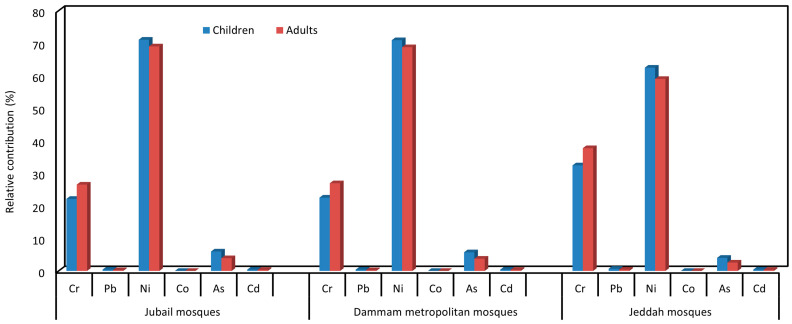
Relative contribution of the individual PHE cancer risk (CR) to ∑CR for total PHEs in Jubail, Dammam metropolitan, and Jeddah mosques for children and adults.

Based on the CR values for the individual PHE, Ni concentrations in the ACFD of mosques displayed higher carcinogenic risks compared with other elements for both children and adults, representing 71.1%, 70.9%, and 62.5% (children) and 69.1%, 68.8%, and 59.0% (adults) of the ∑CR for the ∑PTEs in the JUMs, DMMs, and JEMs, respectively (Figure 8). Higher contributions of Ni to cancer risk were also reported in the indoor dust of Jeddah [21]. The values of ILCRing, ILCRinh, ILCRdermal, and CR for the individual PTEs and ∑PHEs in the mosques of the three studied cities (Table 3, Table 4 and Table 5) were lower and/or within the acceptable level (1 × 10^−6^ to 1 × 10^−4^) [130].

Although the present study deduced that there are no serious non-carcinogenic and carcinogenic risks from exposure to PHEs in mosques’ dust through the different exposure pathways, PHEs exposure can cause serious health effects [71,131,132] by their accumulation in body tissues [133,134], even their HI values at safe levels [131,132]. Accordingly, it can be proposed that PHEs associated with mosques’ dust evaluation should be conducted regularly to ensure lower health risks. The output of the present study may give the initial indication of the risk from PHEs exposure in Saudi Arabian mosques.

In order to minimize the effects of PHEs’ pollution and keep the indoor air quality of the mosques, some precautions and recommendations need to be implemented. These are to keep both doors and windows of the mosques firmly closed especially on windy days, regular cleaning, and the maintenance of the ventilation system, impose a shoes-taking-off strategy, use the slush scraper/dust-adsorbing carpet at the main entrances of the indoor environments [94], and use of wet wiping instead of dry wiping methods to clean up the dust from indoor environments [135].

## 4. Conclusions

In the present study, the concentrations of potentially harmful elements (PHEs) were determined in dust collected from the AC filters of the mosques in three cities of Saudi Arabia. The concentrations of all selected PHEs were found to follow the following order: Fe > Al > Zn > Mn > Cu > Pb > Cr > Ni > V > Co > As > Cd. The total concentrations of all the PHEs in the ACDF of mosques were found to be higher than the background concentration in three cities and following the order of JEM > DMM > JUM, (1407, 1239, 1103 mg/kg), respectively, reflecting the influence of urbanization activities, traffic density, and economic growth on airborne pollution.

The average concentrations of As, Cd, Cr, Cu, Ni, Pb, and Zn were higher than the local soil concentration in these mosques indicating that anthropogenic sources affected the air quality and Pb, Cu, and Zn exceeded the permissible limits. Similarly, the concentrations of the crustal elements (Fe, Al, and Mn) were also found to be higher than the local background concentrations. The spatial distribution of non-crustal elements at different locations of the mosques generally follows the order WSA > SA > UA (1446, 1188, 1113 mg/kg, respectively). Although it is not consistent, it is however correlated with the local population and traffic density.

Based on the pollution-load index classification criteria and cumulative load impact in comparison with the global crustal and local background soil data showed a high pollution load of PHE in all the mosques and were found in the order JEM > DMM > JUM (2378.5, 563.5, and 349.9 PLI based on local background concentration). The highest pollution load of all the PHEs in Jeddah and Dammam may be due to high traffic emissions around the mosques.

The I_geo_ accumulation Index based on global crustal and local background indicated that the highest values were found for Cd, Cu, Pb, and Zn (2.25, 2.82, 2.56, and 2.99, respectively) and were lowest for Co, Cr, Fe, Mn, Ni, and V (−3.06, −1.73, −2.94, −2.39, −1.36, and −2.84, respectively). Based on the I_geo_ index compared to global crustal and local background data, the ACFD were categorized as unpolluted by Fe, Mn, Co, Cr, and V; uncontaminated to moderately contaminated by As, and Cu; moderately contaminated by Cd, Cu, and Pb; and moderately contaminated by Zn at various locations.

The most important findings of this study are the ecological risk assessment, human health risk, and carcinogenic risk evaluation. The highest ER value was calculated for Cd, As, and Pb in JUM, DMM, and JEM, respectively. Based on the ecological risk criteria, Cd was found to have a very high potential for ecological risk, while Pb had a moderate risk. The higher ERI was found in JEM whereas the lowest value was observed in JUM. The non-carcinogenic health risks of the ACFD on children and adults were evaluated for ingestion, inhalation, and dermal contact, and it was estimated that ingestion for both adult and children was the main exposure pathway of PHEs associated with the ACFD of the JUMs, DMMs, and JEMs. The ILCR_ing_ for children was 1.4 times higher than that of adults, whereas ILCR_inh_ and ILCR_dermal_ for adults were 2.4 and 13.7 times higher than that of children in the various mosques. The CR for adults was 1.3 times higher than that for children, confirming that exposure to indoor mosques dust can cause more potential health risks for adults than for children. This difference in cancer risks among the mosques of the three cities suggested the considerable effects of source emissions of PHEs on health risk.

To improve indoor air quality and to protect human exposure to various pollutants, an efficient AC filter with regular cleaning and maintenance or having positive pressure inside the mosque is recommended. Moreover, keeping the door closed or an intermediary lobby between the main entrances and the mosques’ prayer halls can reduce the chances of allowing outdoor air to enter the mosques.

## Figures and Tables

**Table 1:** Descriptive statistics of PHEs content in ACFD of the mosques in Jubail, Dammam metropolitan, and Jeddah cities.

City		Fe	Al	Zn	Mn	Cu	Cr	Pb	Ni	V	Co	As	Cd
Jubail	Minimum (mg/kg)	7831.0	3368.5	362.2	270.7	95.3	33.5	26.6	32.4	19.6	3.2	2.2	0.7
	Maximum (mg/kg)	15,006.0	11,005.5	1065.1	478.0	273.6	57.3	97.5	55.7	40.8	8.1	6.0	1.7
	Median (mg/kg)	11,116.0	4932.5	768.1	370.0	131.7	47.1	33.8	45.5	28.3	4.1	4.6	1.0
	Arithmetical mean (AM) (mg/kg)	10,797.9	5662.8	764.1	386.0	160.9	45.1	51.2	43.7	28.5	4.6	4.3	1.1
	Geometrical mean (GM) (mg/kg)	10,503.1	5248.4	731.8	381.1	150.2	44.3	44.8	42.8	27.6	4.4	4.1	1.0
	Standard deviation (SD)	2679.3	2519.5	219.8	64.3	67.3	8.6	28.1	9.3	7.8	1.7	1.3	0.3
	Standard Error (SE)	893.1	839.8	73.3	21.4	22.4	2.9	9.4	3.1	2.6	0.6	0.4	0.1
	Coefficient of variation (CV, %)	24.8	44.5	28.8	16.7	41.8	19.1	55.0	21.3	27.3	36.1	29.4	32.1
	95% confidence limit value (C95%UCL)	12,857.0	7598.0	932.9	435.4	212.6	51.7	72.8	50.8	34.5	5.9	5.3	1.4
Dammam metropolitan	Minimum (mg/kg)	6611.0	4063.5	365.0	143.3	109.3	36.1	27.9	29.1	17.2	4.0	2.4	0.8
	Maximum (mg/kg)	17,356.0	11,305.5	1672.1	401.8	340.5	99.0	171.9	68.4	43.8	8.8	7.6	1.8
	Median (mg/kg)	11,426.0	7675.5	781.0	284.1	222.9	47.7	40.0	46.2	33.8	6.4	4.6	1.2
	Arithmetical mean (AM) (mg/kg)	11,138.2	7469.0	832.3	271.5	208.6	50.6	54.3	48.1	32.7	6.6	4.5	1.2
	Geometrical mean (GM) (mg/kg)	10,871.9	7303.7	765.8	265.2	202.0	49.2	47.1	47.2	31.8	6.4	4.3	1.2
	Standard deviation (SD)	2540.4	1556.0	357.0	57.5	52.8	14.3	37.1	9.2	7.2	1.3	1.5	0.3
	Standard Error (SE)	598.8	366.8	84.2	13.6	12.5	3.4	8.7	2.2	1.7	0.3	0.4	0.1
	Coefficient of variation (CV, %)	22.8	20.8	42.9	21.2	25.3	28.2	68.3	19.1	21.9	20.0	33.4	25.8
	95% confidence limit value (C95%UCL)	12,401.5	8077.5	1009.9	300.1	234.9	57.7	72.7	52.6	36.2	7.2	5.3	1.4
Jeddah	Minimum (mg/kg)	7911.0	5318.5	464.0	186.7	123.3	37.0	60.7	31.3	40.7	5.4	1.3	1.0
	Maximum (mg/kg)	29,246.0	16,055.5	1774.1	530.8	514.6	668.8	298.3	91.1	95.4	24.6	5.8	2.1
	Median (mg/kg)	14,496.0	9992.5	751.7	320.2	173.6	47.0	84.0	41.5	58.7	9.0	3.6	1.4
	Arithmetical mean (AM) (mg/kg)	15,508.1	10,129.1	835.7	347.0	214.4	84.8	110.2	49.3	60.1	10.3	3.7	1.4
	Geometrical mean (GM) (mg/kg)	14,824.1	9815.4	789.4	335.3	196.6	58.2	97.6	47.4	58.2	9.7	3.6	1.4
	Standard deviation (SD)	5000.6	2628.9	314.0	93.0	106.0	134.2	64.7	15.7	15.8	4.2	1.0	0.3
	Standard Error (SE)	1066.1	560.5	66.9	19.8	22.6	28.6	13.8	3.3	3.4	0.9	0.2	0.1
	Coefficient of variation (CV, %)	32.2	26.0	37.6	26.8	49.5	158.3	58.7	31.8	26.3	41.3	27.9	20.8
	95% confidence limit value (C95%UCL)	17,725.2	11,294.7	974.9	388.2	261.4	144.3	138.9	56.3	67.1	12.2	4.2	1.6
Crustal background value [42,43]		56,300.0	82,300.0	70.0	950.0	55.0	100.0	12.5	75.0	135.0	25.0	1.8	0.2
Local background soil value [45]		18,500.0	-	81.0	520.0	20.2	41.0	15.2	22.0	34.9	4.6	2.3	0.4

**Table 2:** Comparison of the PHEs concentrations (mg/kg) in the ACFD from mosques in Saudi Arabia with indoor dust from other different cities of the world.

Location	Concentration	Fe	Al	Zn	Mn	Cu	Pb	Cr	Ni	As	Co	V	Cd	Reference
Saudi Arabia (Jubail)	Average	10,797.9	5662.8	764.1	386.0	160.9	51.2	45.1	43.7	4.3	4.6	28.5	1.1	Present study
	Median	11,116.0	4932.5	768.1	370.0	131.7	33.8	47.1	45.5	4.6	4.1	28.3	1.0	
Saudi Arabia (Dammam metropolitan)	Average	11,138.2	7469.0	832.3	271.5	208.6	54.3	50.6	48.1	4.5	6.6	32.7	1.2	Present study
	Median	11,426.0	7675.5	781.0	284.1	222.9	40.0	47.7	46.2	4.6	6.4	33.8	1.2	
Saudi Arabia (Jeddah)	Average	15,508.1	10,129.1	835.7	347.0	214.4	110.2	84.8	49.3	3.7	10.3	60.1	1.4	Present study
	Median	14,496.0	9992.5	751.7	320.2	173.6	84.0	47.0	41.5	3.6	9.0	58.7	1.4	
Kuwait	Average	14,453	12,697	784.0	441.0	209.0	158.0	90.0	56.0	13.0	12.5			Al-Harbi et al. [67]
Slovenia (Maribor)	Median	-	-	716.0	306.0	140.0	69.0	65.0	38.0	4.1	6.2	17.0	1.2	Gabersek and Gosar [86]
Canada (Toronto)	Median	-	-	386.0	58.0	136.0	36.0	42.0	23.0	-	-	-	1.7	Al Hejami et al. [87]
China (Tongling)	Average	27,111.2	-	978.9	-	323.1	279.2	62.3	52.7	-	4.3	-	5.1	Yangbing et al. [88]
Pakistan (Lahore)	Average	-	-	1053.0	-	116.0	230.0	194.0	53.6	7.5	-	36.7	5.2	Rehman et al. [89]
Nepal (viz. Kathmandu, Pokhara, Birgunj, and Biratnagar)	Median	838.0	-	1260.0	1650.0	275.0	233.0	231.0	122.0	3.0	28.1	-	1.8	Yadav et al. [90]
Iran (Shiraz)	Average	16,945.5	-	258.8	288.9	40.0	258.8	172.8	50.1	2.8	6.4	-	1.0	Moghtaderi et al. [91]
Saudi Arabia (Jeddah)	Average	-	-	524.0	270.0	126.0	-	61.4	43.2	-	10.7	-	0.9	Albar et al. [75]
Qatar (Doha city)	Average	20,504	19,812	824.3	369.8	192.9	65.3	91.8	68.7	7.2	12.3	52.1	0.7	Mahfouz et al. [92]
Saudi Arabia (Jeddah)	Average	8752.0	-	343.0	392.0	88.0	121.0	40.0	36.0	8.0	8.1	43.9	2.1	Alghamdi et al. [21]
Nigeria (southern Nigeria)	Median	11,300	5430.0	1110.0	161.0	161.0	207.0	40.1	25.3	-	3.8	-	3.8	Iwegbue et al. [93]
Australia (Sydney)	Average	-	-	1876.0	220.0	272.0	299.0	90.0	50.9	17.6	-	-		Doyi et al. [94]
Jordan (Jordanian)	Average	8400.0	700.0	4500.0	-	500.0	-	90.0	40.0	-	2.0	-	5.0	Arar et al. [95]
Portugal (Estarreja)	Mean	-	11,500	2090.0	188.0	311.0	380.0	63.0	13.0	13.0	5.1	18.0	1.4	Plumejeaud et al. [96]
Iran (Shiraz Shiraz megacity)	Average	-	-	233.3	-	40.0	258.9	172.8	50.1	2.8	6.4	-	1.0	Moghtaderi et al. [97]
Saudi Arabia (Riyadh)	Average	-	-	94.4	90.0	71.8	24.8	27.0	20.3	-	2.0	15.4	-	El-Mubarak et al. [98]
Egypt (Cairo)	Average	20,818.0	9092.0	419.0	425.0	116.6	99.3	48.6	30.1	4.0	9.0	45.7	1.0	Gad et al. [99]

**Table 3:** Summary of hazard quotients (HQ), hazard index (HI), incremental lifetime cancer risk (ILCR), and cancer risk (CR) of PHEs in ACFD collected samples from Jubail mosques via non-dietary ingestion, inhalation, and dermal contact exposure pathways.

Non-Cancer Risks	Children				Adults			
PHEs	HQ_ing_	HQ_inh_	HQ_dermal_	HI	HQ_ing_	HQ_inh_	HQ_dermal_	HI
Fe	1.8 × 10^−3^	1.9 × 10^−3^	3.4 × 10^−4^	4.0 × 10^−3^	1.9 × 10^−4^	6.9 × 10^−4^	7.0 × 10^−4^	1.6 × 10^−3^
Al	7.9 × 10^−3^	4.4 × 10^−7^	1.3 × 10^−5^	7.9 × 10^−3^	8.4 × 10^−4^	1.6 × 10^−7^	2.6 × 10^−5^	8.7 × 10^−4^
Zn	3.5 × 10^−3^	9.9 × 10^−8^	2.8 × 10^−5^	3.6 × 10^−3^	3.8 × 10^−4^	3.6 × 10^−8^	5.8 × 10^−5^	4.4 × 10^−4^
Mn	1.1 × 10^−2^	1.1 × 10^−3^	4.7 × 10^−4^	1.3 × 10^−2^	1.2 × 10^−3^	3.8 × 10^−4^	9.5 × 10^−4^	2.6 × 10^−3^
Cu	5.6 × 10^−3^	1.6 × 10^−7^	3.0 × 10^−5^	5.6 × 10^−3^	6.0 × 10^−4^	5.6 × 10^−8^	6.1 × 10^−5^	6.6 × 10^−4^
Cr	2.1 × 10^−2^	6.1 × 10^−5^	2.0 × 10^−3^	2.3 × 10^−2^	2.2 × 10^−3^	2.2 × 10^−5^	4.1 × 10^−3^	6.3 × 10^−3^
Pb	2.0 × 10^−2^	5.7 × 10^−7^	2.2 × 10^−4^	2.1 × 10^−2^	2.2 × 10^−3^	2.0 × 10^−7^	4.4 × 10^−4^	2.6 × 10^−3^
Ni	3.0 × 10^−3^	8.3 × 10^−8^	1.8 × 10^−5^	3.1 × 10^−3^	3.2 × 10^−4^	3.0 × 10^−8^	3.7 × 10^−5^	3.6 × 10^−4^
V	5.7 × 10^−3^	1.6 × 10^−7^	9.1 × 10^−4^	6.6 × 10^−3^	6.1 × 10^−4^	5.7 × 10^−8^	1.8 × 10^−3^	2.5 × 10^−3^
Co	3.2 × 10^−4^	3.1 × 10^−5^	6.4 × 10^−7^	3.5 × 10^−4^	3.4 × 10^−5^	1.1 × 10^−5^	1.3 × 10^−6^	4.7 × 10^−5^
As	2.0 × 10^−2^	5.6 × 10^−7^	7.8 × 10^−5^	2.0 × 10^−2^	2.1 × 10^−3^	2.0 × 10^−7^	1.6 × 10^−4^	2.3 × 10^−3^
Cd	1.5 × 10^−3^	4.2 × 10^−8^	2.4 × 10^−4^	1.8 × 10^−3^	1.6 × 10^−4^	1.5 × 10^−8^	4.9 × 10^−4^	6.5 × 10^−4^
Total	1.0 × 10^−1^	3.1 × 10^−3^	4.3 × 10^−3^	1.1 × 10^−1^	1.1 × 10^−2^	1.1 × 10^−3^	8.9 × 10^−3^	2.1 × 10^−2^
**Cancer Risks**	**Children**				**Adults**			
**PHEs**	**ILCR_ing_**	**ILCR_inh_**	**ILCR_dermal_**	**CR**	**ILC_Ring_**	**ILCR_inh_**	**ILCR_dermal_**	**CR**
Cr	1.27 × 10^−6^	2.97 × 10^−9^	8.09 × 10^−8^	1.4 × 10^−6^	9.10 × 10^−7^	7.18 × 10^−9^	1.11 × 10^−6^	2.0 × 10^−6^
Pb	2.44 × 10^−8^	3.37 × 10^−12^	3.90 × 10^−11^	2.4 × 10^−8^	1.75 × 10^−8^	8.15 × 10^−12^	5.33 × 10^−10^	1.8 × 10^−8^
Ni	4.17 × 10^−6^	5.76 × 10^−11^	1.67 × 10^−7^	4.3 × 10^−6^	2.99 × 10^−6^	1.39 × 10^−10^	2.28 × 10^−6^	5.3 × 10^−6^
Co		7.08 × 10^−11^		7.1 × 10^−11^		1.71 × 10^−10^		1.7 × 10^−10^
As	3.62 × 10^−7^	1.01 × 10^−11^	2.89 × 10^−9^	3.6 × 10^−7^	2.59 × 10^−7^	2.44 × 10^−11^	3.95 × 10^−8^	3.0 × 10^−7^
Cd	2.32 × 10^−8^	6.49 × 10^−13^	5.95 × 10^−10^	2.4 × 10^−8^	1.66 × 10^−8^	1.57 × 10^−12^	8.13 × 10^−9^	2.5 × 10^−8^
Total	5.9 × 10^−6^	3.1 × 10^−9^	2.5 × 10^−7^	6.1 × 10^−6^	4.2 × 10^−6^	7.5 × 10^−9^	3.4 × 10^−6^	7.6 × 10^−6^

**Table 4:** Summary of hazard quotients (HQ), hazard index (HI), incremental lifetime cancer risk (ILCR), and cancer risk (CR) of PHEs in ACFD collected samples from Dammam metropolitan mosques via non-dietary ingestion, inhalation, and dermal contact exposure pathways.

Non-Cancer Risks	Children				Adults			
PHEs	HQ_ing_	HQ_inh_	HQ_dermal_	HI	HQ_ing_	HQ_inh_	HQ_dermal_	HI
Fe	1.8 × 10^−3^	2.0 × 10^−3^	3.5 × 10^−4^	4.2 × 10^−3^	2.0 × 10^−4^	7.1 × 10^−4^	7.2 × 10^−4^	1.6 × 10^−3^
Al	1.0 × 10^−2^	5.8 × 10^−7^	1.7 × 10^−5^	1.0 × 10^−2^	1.1 × 10^−3^	2.1 × 10^−7^	3.4 × 10^−5^	1.1 × 10^−3^
Zn	3.9 × 10^−3^	1.1 × 10^−7^	3.1 × 10^−5^	3.9 × 10^−3^	4.1 × 10^−4^	3.9 × 10^−8^	6.3 × 10^−5^	4.8 × 10^−4^
Mn	8.0 × 10^−3^	7.4 × 10^−4^	3.3 × 10^−4^	9.1 × 10^−3^	8.6 × 10^−4^	2.7 × 10^−4^	6.7 × 10^−4^	1.8 × 10^−3^
Cu	7.2 × 10^−3^	2.0 × 10^−7^	3.9 × 10^−5^	7.3 × 10^−3^	7.8 × 10^−4^	7.3 × 10^−8^	7.9 × 10^−5^	8.5 × 10^−4^
Cr	2.3 × 10^−2^	6.9 × 10^−5^	2.2 × 10^−3^	2.6 × 10^−2^	2.5 × 10^−3^	2.5 × 10^−5^	4.6 × 10^−3^	7.1 × 10^−3^
Pb	2.2 × 10^−2^	6.0 × 10^−7^	2.3 × 10^−4^	2.2 × 10^−2^	2.3 × 10^−3^	2.2 × 10^−7^	4.7 × 10^−4^	2.8 × 10^−3^
Ni	3.3 × 10^−3^	9.1 × 10^−8^	2.0 × 10^−5^	3.4 × 10^−3^	3.6 × 10^−4^	3.3 × 10^−8^	4.0 × 10^−5^	4.0 × 10^−4^
V	6.5 × 10^−3^	1.8 × 10^−7^	1.0 × 10^−3^	7.5 × 10^−3^	6.9 × 10^−4^	6.5 × 10^−8^	2.1 × 10^−3^	2.8 × 10^−3^
Co	4.6 × 10^−4^	4.5 × 10^−5^	9.1 × 10^−7^	5.0 × 10^−4^	4.9 × 10^−5^	1.6 × 10^−5^	1.9 × 10^−6^	6.7 × 10^−5^
As	2.1 × 10^−2^	5.9 × 10^−7^	8.2 × 10^−5^	2.1 × 10^−2^	2.3 × 10^−3^	2.1 × 10^−7^	1.7 × 10^−4^	2.4 × 10^−3^
Cd	1.7 × 10^−3^	4.7 × 10^−8^	2.7 × 10^−4^	2.0 × 10^−3^	1.8 × 10^−4^	1.7 × 10^−8^	5.5 × 10^−4^	7.3 × 10^−4^
Total	1.1 × 10^−1^	2.8 × 10^−3^	4.7 × 10^−3^	1.2 × 10^−1^	1.2 × 10^−2^	1.0 × 10^−3^	9.5 × 10^−3^	2.2 × 10^−2^
**Cancer Risks**	**Children**				**Adults**			
**PHEs**	**ILCR_ing_**	**ILCR_inh_**	**ILCR_dermal_**	**CR**	**ILCR_ing_**	**ILCR_inh_**	**ILCR_dermal_**	**CR**
Cr	1.42 × 10^−6^	3.34 × 10^−9^	9.07 × 10^−8^	1.5 × 10^−6^	1.02 × 10^−6^	8.05 × 10^−9^	1.24 × 10^−6^	2.3 × 10^−6^
Pb	2.59 × 10^−8^	3.58 × 10^−12^	4.14 × 10^−11^	2.6 × 10^−8^	1.86 × 10^−8^	8.64 × 10^−12^	5.65 × 10^−10^	1.9 × 10^−8^
Ni	4.59 × 10^−6^	6.34 × 10^−11^	1.83 × 10^−7^	4.8 × 10^−6^	3.29 × 10^−6^	1.53 × 10^−10^	2.50 × 10^−6^	5.8 × 10^−6^
Co		1.01 × 10^−10^		1.0 × 10^−10^		2.44 × 10^−10^		2.4 × 10^−10^
As	3.83 × 10^−7^	1.07 × 10^−11^	3.06 × 10^−9^	3.9 × 10^−7^	2.75 × 10^−7^	2.59 × 10^−11^	4.18 × 10^−8^	3.2 × 10^−7^
Cd	2.59 × 10^−8^	7.22 × 10^−13^	6.63 × 10^−10^	2.7 × 10^−8^	1.85 × 10^−8^	1.74 × 10^−12^	9.06 × 10^−9^	2.8 × 10^−8^
Total	6.5 × 10^−6^	3.5 × 10^−9^	2.8 × 10^−7^	6.7 × 10^−6^	4.6 × 10^−6^	8.5 × 10^−9^	3.8 × 10^−6^	8.4 × 10^−6^

**Table 5:** Summary of hazard quotients (HQ), hazard index (HI), incremental lifetime cancer risk (ILCR), and cancer risk (CR) of PHEs in ACFD collected samples from Jeddah mosques via non-dietary ingestion, inhalation, and dermal contact exposure pathways.

Non-Cancer Risks	Children				Adults			
PHEs	HQ_ing_	HQ_inh_	HQ_dermal_	HI	HQ_ing_	HQ_inh_	HQ_dermal_	HI
Fe	2.6 × 10^−3^	2.7 × 10^−3^	4.9 × 10^−4^	5.8 × 10^−3^	2.7 × 10^−4^	9.9 × 10^−4^	1.0 × 10^−3^	2.3 × 10^−3^
Al	1.4 × 10^−2^	7.9 × 10^−7^	2.3 × 10^−5^	1.4 × 10^−2^	1.5 × 10^−3^	2.8 × 10^−7^	4.6 × 10^−5^	1.6 × 10^−3^
Zn	3.9 × 10^−3^	1.1 × 10^−7^	3.1 × 10^−5^	3.9 × 10^−3^	4.1 × 10^−4^	3.9 × 10^−8^	6.3 × 10^−5^	4.8 × 10^−4^
Mn	1.0 × 10^−2^	9.5 × 10^−4^	4.2 × 10^−4^	1.2 × 10^−2^	1.1 × 10^−3^	3.4 × 10^−4^	8.5 × 10^−4^	2.3 × 10^−3^
Cu	7.4 × 10^−3^	2.1 × 10^−7^	4.0 × 10^−5^	7.5 × 10^−3^	8.0 × 10^−4^	7.5 × 10^−8^	8.1 × 10^−5^	8.8 × 10^−4^
Cr	3.9 × 10^−2^	1.2 × 10^−4^	3.8 × 10^−3^	4.3 × 10^−2^	4.2 × 10^−3^	4.2 × 10^−5^	7.7 × 10^−3^	1.2 × 10^−2^
Pb	4.4 × 10^−2^	1.2 × 10^−6^	4.7 × 10^−4^	4.4 × 10^−2^	4.7 × 10^−3^	4.4 × 10^−7^	9.5 × 10^−4^	5.6 × 10^−3^
Ni	3.4 × 10^−3^	9.3 × 10^−8^	2.0 × 10^−5^	3.4 × 10^−3^	3.7 × 10^−4^	3.4 × 10^−8^	4.1 × 10^−5^	4.1 × 10^−4^
V	1.2 × 10^−2^	3.3 × 10^−7^	1.9 × 10^−3^	1.4 × 10^−2^	1.3 × 10^−3^	1.2 × 10^−7^	3.9 × 10^−3^	5.2 × 10^−3^
Co	7.1 × 10^−4^	7.0 × 10^−5^	1.4 × 10^−6^	7.8 × 10^−4^	7.6 × 10^−5^	2.5 × 10^−5^	2.9 × 10^−6^	1.0 × 10^−4^
As	1.7 × 10^−2^	4.8 × 10^−7^	6.7 × 10^−5^	1.7 × 10^−2^	1.8 × 10^−3^	1.7 × 10^−7^	1.4 × 10^−4^	2.0 × 10^−3^
Cd	2.0 × 10^−3^	5.6 × 10^−8^	3.2 × 10^−4^	2.3 × 10^−3^	2.1 × 10^−4^	2.0 × 10^−8^	6.5 × 10^−4^	8.6 × 10^−4^
Total	1.6 × 10^−1^	3.9 × 10^−3^	7.6 × 10^−3^	1.7 × 10^−1^	1.7 × 10^−2^	1.4 × 10^−3^	1.5 × 10^−2^	3.4 × 10^−2^
**Cancer Risks**	**Children**				**Adults**			
**PHEs**	**ILCR_ing_**	**ILCR_inh_**	**ILCR_dermal_**	**CR**	**ILCR_ing_**	**ILCR_inh_**	**ILCR_derma_l**	**CR**
Cr	2.39 × 10^−6^	5.59 × 10^−9^	1.52 × 10^−7^	2.5 × 10^−6^	1.71 × 10^−6^	1.35 × 10^−8^	2.08 × 10^−6^	3.8 × 10^−6^
Pb	5.26 × 10^−8^	7.26 × 10^−12^	8.40 × 10^−11^	5.3 × 10^−8^	3.77 × 10^−8^	1.75 × 10^−11^	1.15 × 10^−9^	3.9 × 10^−8^
Ni	4.71 × 10^−6^	6.51 × 10^−11^	1.88 × 10^−7^	4.9 × 10^−6^	3.38 × 10^−6^	1.57 × 10^−10^	2.57 × 10^−6^	5.9 × 10^−6^
Co		1.58 × 10^−10^		1.6 × 10^−10^		3.82 × 10^−10^		3.8 × 10^−10^
As	3.14 × 10^−7^	8.77 × 10^−12^	2.51 × 10^−9^	3.2 × 10^−7^	2.25 × 10^−7^	2.12 × 10^−11^	3.42 × 10^−8^	2.6 × 10^−7^
Cd	3.06 × 10^−8^	8.54 × 10^−13^	7.83 × 10^−10^	3.1 × 10^−8^	2.19 × 10^−8^	2.06 × 10^−12^	1.07 × 10^−8^	3.3 × 10^−8^
Total	7.5 × 10^−6^	5.8 × 10^−9^	3.4 × 10^−7^	7.8 × 10^−6^	5.4 × 10^−6^	1.4 × 10^−8^	4.7 × 10^−6^	1.0 × 10^−5^

## Data Availability

The data that support the findings of this study are available on request from the corresponding author.

## Supplementary Materials

The following supporting information can be downloaded at: https://www.mdpi.com/article/10.3390/ijerph20032687/s1, Table S1: Values of risk parameters used for calculating HQs and ILCRs for children and adults exposed to PHEs associated with ACFD of mosques; Table S2: Reference Doses (R_f_D, mg kg^−1^ day^−1^) of different PHEs used to calculate HQs; Table S3: Carcinogenic Slope Factor (CSF, (mg kg^−1^ day^−1^)^−1^) of different PTEs used to calculate CR [19]; Table S4: Geo-accumulation index (Igeo), contamination factor (Cf), degree of contamination (Cd) and pollution load index (PLI) of PHEs in ACFD of Jubail, Dammam metropolitan and Jeddah mosques; Table S5: Classification values and qualitative description of enrichment factor, geo-accumulation index, contamination factor, and ecological risk indices. Refs [136,137,138,139,140,141,142,143,144,145,146,147,148,149,150,151,152,153,154,155,156,157,158] are cited in Supplementary Materials.
