# Supplementary material for: Potentially Harmful Elements Associated with Dust of Mosques: Pollution Status, Sources, and Human Health Risks
Source: Int J Environ Res Public Health. 2023 Feb 2;20(3):2687. doi: 10.3390/ijerph20032687 (PMC9916264; doi:10.3390/ijerph20032687)
Supplement: Supplementary file 1 [file ijerph-20-02687-s001.zip › ijerph-2138854-supplementary.pdf]

SUPPLEMENTARY INFORMATION

Table S1: Values of risk parameters used for calculating HQs and ILCRs for children and adults exposed to PHEs associated with ACFD of mosques.

Factor	Description	Value		References
		Children	Adults	
C	Concentration of PHEs in dusts (mg/kg)			Present study
IngR	Ingestion rate of dust (mg/day)	200	100	USEPA [1]; Gope et al. [2]
EF	Exposure frequency (days/year)	(2.5h/24h)*350 =38.02 day/year)	(2.5h/24h)*350 =38.02 day/year)	Peng et al. [3]; Zheng et al. [4]; ESAG [5]
ED	Exposure duration (years)	6	24	USEPA [6]; USEPA [7,8]
BW	Average body weight (kg)	15	70	Hu et al. [9]; Lu et al. [10]; USEPA [11]
AT	Average time (non-carcinogenic effects) (days)	ED x 365	ED x 365	USEPA [12]
AT	Average time (carcinogenic effects) (days)	70 yr ×365	70 yr ×365	USEPA [12]
CF	Conversion factor (kg/mg)	1×10^{-6}	1×10^{-6}	Li et al. (2011)
InhR	Inhalation rate of dust (m^3/day)	7.63	12.8	Li et al. [13]; USEPA [6]
PEF	Particular emission factor (m^3/kg)	1.36×10^9	1.36×10^9	USEPA [7,8]
SA	Exposed surface area of skin (cm^2)	1600	4350	Zheng et al. [4]; ESAG [5]
AF	Skin adherence factor (mg/cm^2)	0.2	0.7	USEPA [1]
ABF	Absorption factor (Dermal)	0.001	0.001	Wei et al. [14]; USEPA [7,8]

Table S2: Reference Doses (R_fD, mg kg^{-1} day^{-1}) of different PHEs used to calculate HQs.

	Fe	Mn	Zn	Pb	Cd	V	Co.	Ni	As	Cr	Cu	References
$Rf D_{ing}$	8.40E+00	4.70E−02	3.00E−01	3.50E−03	1.00E−03	7.00E−03	2.00E−02	2.00E−02	3.00E−04	3.00E−03	4.00E−02	Ali et al., [15]; Shabbaj et al., [16],
$Rf D_{inh}$	2.20E−04	1.43E−05	3.00E−01	3.52E−03	1.00E−03	7.00E−03	5.71E−06	2.06E−02	3.01E−04	2.86E−05	4.02E−02	Li et al., [17];
$Rf D_{dermal}$	7.00E−02	1.84E−03	6.00E−02	5.25E−04	1.00E−05	7.00E−05	1.60E−02	5.40E−03	1.23E−04	5.00E−05	1.20E−02	Alghamdi et al., [18]

Table S3: Carcinogenic Slope Factor (CSF, (mg kg^{-1} day^{-1})$^{-1}$) of different PTEs used to calculate CR [19].

	CSF			References
	$CSF_{Ing.}$	$CSF_{Inh.}$	CSF_{Dermal}	
Cr	0.50	42.00	20.00	Zheng et al. [20]; Kamunda et al. [21]; Ying et al. [22]; Adimalla [23]
Ni	1.70	0.84	42.50	Huang et al. [24]; Lu et al. [25]; Adimalla [23]
Cd	0.38	0.38	6.10	Lu et al. [25]; Kamunda et al. [21]
Pb	0.01	0.04	0.01	Pavilonis et al. [26]; Kamunda et al. [21]; Bello et al. [27]; Johnbull et al. [28]
Co	-	9.80	-	Lu et al. [25]; Zheng et al. [20]; Bello et al. [27]
As	1.50	1.50	7.50	Lu et al. [25]; Ying et al. [22]; Pavilonis et al. [26]

Table S4. Geo-accumulation index (I_{geo}), contamination factor (C_f), degree of contamination (C_d) and pollution load index (*PLI*) of PHEs in ACFD of Jubail, Dammam metropolitan and Jeddah mosques

		Potentially harmful elements (PHEs)										
		As	Cd	Co	Cr	Cu	Fe	Mn	Ni	Pb	V	Zn
Jubail mosques	I_{geo}^a	0.67	1.86	-3.06	-1.73	0.96	-2.94	-1.89	-1.36	1.44	-2.84	2.86
	I_{geo}^b	0.35	0.86	-0.59	-0.45	2.41	-1.36	-1.01	0.40	1.17	-0.87	2.65
Dammam metropolitan mosques	I_{geo}^a	0.75	2.01	-2.47	-1.55	1.34	-2.94	-2.39	-1.21	1.53	-2.64	2.99
	I_{geo}^b	0.43	1.01	-0.07	-0.28	2.78	-1.32	-1.52	0.54	1.25	-0.68	2.78
Jeddah mosques	I_{geo}^a	0,46	2.25	-1.89	-0.81	1.38	-2.47	-2.06	-1.18	2.56	-1.74	2.99
	I_{geo}^b	0.14	1.25	0.57	0.46	2.82	-0.84	-1.17	0.58	2.27	0.20	2.78
Jubail mosques	C_f^a	2.39	5.44	0.18	0.45	2.93	0.19	0.41	0.58	4.09	0.21	10.92
	C_f^b	1.91	2.72	1.00	1.10	7.96	0.58	0.74	1.99	3.37	0.82	9.43
Dammam metropolitan mosques	C_f^a	2.53	6.06	0.26	0.51	3.79	0.20	0.29	0.64	4.34	0.24	11.89
	C_f^b	2.02	3.03	1.43	1.23	10.33	0.60	0.52	2.18	3.57	0.94	10.28
Jeddah mosques	C_f^a	2.07	7.16	0.41	0.85	3.90	0.28	0.37	0.66	8.81	0.45	11.94
	C_f^b	1.66	3.58	2.23	2.07	10.61	0.84	0.67	2.24	7.24	1.72	10.32
Jubail mosques	C_d^a						27.85					
	C_d^b						31.61					
Dammam metropolitan mosques	C_d^a						30.83					
	C_d^b						36.13					
Jeddah mosques	C_d^a						37.00					
	C_d^b						43.17					
Jubail mosques	PLI^a						3.35					
	PLI^b						349.91					
Dammam metropolitan mosques	PLI^a						6.19					
	PLI^b						563.53					
Jeddah mosques	PLI^a						30.47					
	PLI^b						2378.53					

[a] based on the global crustal average data
[b] based on the local background soil

Table S5. Classification values and qualitative description of enrichment factor, geo-accumulation index, contamination factor and ecological risk indices

Pollution and risk indicators	Classification values	Qualitative designation of SW dust
Enrichment factor (EF)[a]	<1	No enrichment
	1–2	Light enrichment
	2–5	Moderate enrichment
	5–20	Significant enrichment
	20–40	Strong enrichment
	>40	Extreme enrichment
Geo-accumulation index (I_{geo})[b]	$I_{geo} \leq 0$	Uncontaminated
	$0 < I_{geo} \leq 1$	Uncontaminated to moderately contaminated
	$1 < I_{geo} \leq 2$	Moderately contaminated
	$2 < I_{geo} \leq 3$	Moderately to heavily contaminated
	$3 < I_{geo} \leq 4$	Heavily contaminated
	$4 < I_{geo} \leq 5$	Heavily to extremely contaminated
	$I_{geo} > 5$	Extremely contaminated
Contamination factor (C_f)[c]	Cf <1	Low contamination
	Cf = (1–3)	Moderate contamination
	Cf = (3–6)	Considerable contamination
	Cf = (>6)	Very high contamination
Contamination degree (C_d)[d]	Cd < 8	Low degree of contamination
	8 < Cd ≤16	Moderate degree of contamination
	16 < Cd≤ 32	Moderate to a strong degree of contamination
	Cd > 32	Very strong degree of contamination
Pollution load index (PLI)[e,f]	PLI <1	No pollution
	1 ≤ PLI < 2	Slight pollution
	2 ≤ PLI < 3	Moderate pollution
	3 ≤ PLI	Heavy pollution
Ecological risk (ER and ERI)[f], [g]	ER<40; ERI<150	Lower potential ecological risk
	40 ≤ER< 80; 150≤ ERI<300	Moderate level of potential ecological risk
	80 ≤ ER<160; 300 ≤ ERI<600	Considerable potential ecological risk
	160≤ ER<320; ERI ≥ 600	Very high potential ecological risk
	ER ≥ 320	Dangerous

[a] Li et al. [29]; [b] Zhang et al. [30]; [c] Liu et al. [31]; [d]Yaylalı-Abanuz [32]; [e] Gope et al. [33]; [f] Mohammadi et al. [34]; [g]Zhang et al. [30]

References

1. United States Environmental Protection, Agency (USEPA). Supplemental Guidance for Developing Soil ScreeningLevels for Superfund Sites; Office of Solid Waste and Emergency Response (OSWER): Washington, DC, USA, 2011.
2. Gope, M., Masto, R.E., George, J., Hoque, R.R., Balachandran, S., 2017. Bioavailability and health risk of some potentially toxic elements (Cd, Cu, Pb and Zn) in street dust of Asansol, India. Ecotoxicology and Environmental Safety, 138, 231–241.
3. Peng, C.; Chen, W.; Liao, X.; Wang, M.; Ouyang, Z.; Jiao, W.; Bai, Y. Polycyclic aromatic hydrocarbons in urban soils of Beijing: Status, sources, distribution and potential risk. Environ. Pollut. 2011, 159, 802–808.
4. Zheng, N., Liu, J., Wang, Q., Liang, Z., 2010. Health risk assessment of heavy metal exposure to street dust in the zinc smelting district, Northeast of China. Sci. Total Environ. 408, 726-733.
5. ESAG. Environmental Site Assessment Guideline; DB11/T656–2009; Adelaide Airport: Adelaide, Australia,2009. (In Chinese).
6. United States Environmental Protection, Agency (USEPA). Child-Specific Exposure Factors Handbook; EPA-600-P-00e002B; National Center for Environmental Assessment: Washington, DC, USA, 2002.
7. United States Environmental Protection, Agency (USEPA). Risk Assessment Guidance for Superfund: Volume III—Part A, Process for Conducting Probabilistic RiskAssessment; EPA540-R-02-002; U.S. Environmental Protection Agency: Washington, DC, USA, 2001a.
8. United States Environmental Protection, Agency (USEPA). Child-Specific Exposure Factors Handbook; EPA-600-P-00-002B; National Center for Environmental Assessment: Washington, DC, USA, 2001b.
9. Hu, X.; Zhang, Y.; Luo, J.; Wang, T.J.; Lian, H.Z.; Ding, Z.H. Bioaccessibility and health risk of ar senic, mercury and other metals in urban street dusts from a mega-city, Nanjing, China. Environ. Pollut. 2011, 159, 1215–1221.
10. Lu, X, Wang, ., Li, LY, Lei, K., Huang, L, Kang, D. Multivariate statistical analysis of heavy metals in street dust of Baoji NW China. J Hazard Mater 2010;173:744-749.
11. United States Environmental Protection, Agency (USEPA). Risk Assessment Guidance for Superfund, Vol.I: Human Health Evaluation Manual (PartA); EPA/540/1-89/002; Officeof Emergency and Remedial Response: Washington, DC, USA,1989
12. USEPA. Risk Assessment Guidance for Superfund. Volume I: Human Health Evaluation Manual (Part E, Supplemental Guidance for Dermal Risk Assessment). EPA/540/R/99; 2004.
13. Li, R.Z.; Zhou, A.J.; Tong, F.; Wu, Y.D.; Zhang, P.; Yu, J. Distribution of metals in urban dusts of Hefei and health risk assessment. Chin. J. Environ. Sci. 2011, 32, 2661–2668.
14. Wei, X., Gao, B., Wang, P., Zhou, H., Lu, J., 2015. Pollution characteristics and health risk assessment of heavy metals in street dusts from different functional areas in Beijing, China. Ecotoxicol. Environ. Saf. 112, 186-192.

15. Ali, MU, Liu, G, Yousaf, B, Abbas, Q, Ullah, H, Munir, MAM, Fu, B. Pollution characteristics and human health risks of potentially (eco) toxic elements (PTEs) in road dust from metropolitan area of Hefei, China. Chemosphere 2017:181:111-121.
16. Shabbaj, II, Alghamdi, MA, Shamy, M, Hassan, SK, Alsharif, MM, Khoder, MI. Risk Assessment and Implication of Human Exposure to Road Dust Heavy Metals in Jeddah, Saudi Arabia. Int J Environ Res Public Health 2018;15:36.
17. Li, H-H, Chen, L-J, Yu, L, Guo, Z-B, Shan, C-Q, Lin, J-Q, Gu, Y-G, Yang, Z-B, Yang, Y-X, Shao, J-R, Zhu, X-M, Cheng, Z. Pollution characteristics and risk assessment of human exposure to oral bioaccessibility of heavy metals via urban street dusts from different functional areas in Chengdu, China. Sci Tot Environ 2017;586:1076-1084.
18. Alghamdi, MA, Hassan, SK, Alzahrani, NA, Almehmadi, FM, Khoder MI. Risk assessment and implications of schoolchildren exposure to classroom heavy metals particles in Jeddah, Saudi Arabia. Int J Environ Res Pub Health 2019;16:5017.
19. Patel, DK, Jain, MK. Contamination and health risk assessment of potentially harmful elements associated with roadside dust in Dhanbad India. Stochastic Environ Res Risk Assess 2022;36:389-407.
20. Zheng, X, Zhao, W, Yan, X, Shu, T, Xiong, Q, Chen, F. Pollution characteristics and health risk assessment of airborne heavy metals collected from Beijing Bus Stations. Intl J Environ Res Pub Health 2015;12:9658-9671.
21. Kamunda, C, Mathuthu, M, Madhuku, M. Health risk assessment of heavy metals in soils from Witwatersrand Gold Mining Basin, South Africa. Intl J Environ Res Public Health 2016;13:663.
22. Ying, L, Shaogang, L, Xiaoyang, C. Assessment of heavy metal pollution and human health risk in urban soils of a coal mining city in East China. Hum Ecol Risk Assess 2016;22:1359-1374.
23. Adimalla, N. Heavy metals contamination in urban surface soils of Medak province, India, and its risk assessment and spatial distribution. Environ Geochem Health 2019;42:59-75.
24. Huang, SL, Li, Q, Xu, DS. Heavy metal pollution in Suzhou urban soils and its health risk assessment. Adv Mater Res 2012;534:244-248.
25. Lu, X, Zhang, X, Li, LY, Chen, H. Assessment of metals pollution and health risk in dust from nursery schools in Xi'an, China. Environ Res 2014;128:27-34.
26. Pavilonis, B, Grassman, J, Johnson, G, Diaz, Y, Caravanos, J. Characterization and risk of exposure to elements from artisanal gold mining operations in the Bolivian Andes. Environ Res 2017;154:1-9.
27. Bello, S, Muhammad, BG, Bature, B. Total excess lifetime cancer risk estimation from enhanced heavy metals concentrations resulting from tailings in Katsina Steel Rolling Mill, Nigeria. J Mater Sci Eng 2017;6:338.
28. Johnbull, O, Abbassi, B, Zytner, RG. Risk assessment of heavy metals in soil based on the geographic information system - Kriging technique in Anka, Nigeria. Environ Eng Res 2019;24:150-158.
29. Li, N., Han, W., Tang, J., Bian, J., Sun, S., Song, T., 2018. Pollution characteristics and human health risks of elements in road dust in Changchun, China. International Journal of Environmental Research and Public Health 15(9), 1843.

30. Zhang, J, Hua, P, Krebs. P. Influences of land use and antecedent dry-weather period on pollution level and ecological risk of heavy metals in road-deposited sediment. Environ Pollut 2017;228:158-68.

31. Liu, E., Wang, X., Liu, H., Liang, M., Zhu, Y., Li, Z., 2019. Chemical speciation, pollution and ecological risk of toxic metals in readily washed off road dust in a megacity (Nanjing), China. Ecotoxicology and Environmental Safety 173:381-92.

32. Yaylalı-Abanuz, G., 2011. Heavy metal contamination of surface soil around Gebze industrial area, Turkey. Microchem J. 99(1), 82-92.

33. Gope, M., Masto. R.E., George. J., Balachandran. S., 2018. Tracing source, distribution and health risk of potentially harmful elements (PHEs) in street dust of Durgapur, India. Ecotoxicol Environ Saf 154, 280-293.

34. Mohammadi A, Hajizadeh Y, Taghipour H, Arani AM, Mokhtari M, Fallahzadeh H., 2018. Assessment of metals in agricultural soil of surrounding areas of Urmia Lake, northwest Iran: A preliminary ecological risk assessment and source identification. Human and Ecological Risk Assessment 24, 2070-2087.
